# Manipulating Liquid‐Liquid Interphase Solute Diffusion for the Construction of Non‐Symmetric Metal‐Quinone Network Nanoarchitectonics and Nano‐Motorization

**DOI:** 10.1002/advs.202503164

**Published:** 2025-07-03

**Authors:** Wenzhe Xu, Yingke Xue, Yang Chen, Ruixu Yang, Shuwei Liu, Yi Liu, Hao Zhang

**Affiliations:** ^1^ Plastic and Reconstructive Surgery The First Hospital of Jilin University Changchun 130021 P. R. China; ^2^ State Key Laboratory of Supramolecular Structure and Materials, College of Chemistry Jilin University Changchun 130012 P. R. China; ^3^ Joint Laboratory of Opto‐Functional Theranostics in Medicine and Chemistry, Institute of Translational Medicine The First Hospital of Jilin University Changchun 130021 P. R. China

**Keywords:** interphase diffusion, liquid‐liquid interface, metal‐quinone networks, nanomotors, nanoprecipitation, non‐symmetric nanoarchitectonics

## Abstract

Non‐symmetric nanoarchitectonics have demonstrated superior performance in nanotherapeutics compared to their symmetric counterparts, particularly in the design of self‐propelled nanodrug delivery systems. However, achieving precise morphological control of non‐symmetric nanoarchitectonics composed of molecular drugs, without the use of templates and surfactants, remains a significant challenge. In this work, a strategy is presented to construct non‐symmetric metal‐quinone network (MQN) nanoarchitectonics with controlled morphology and size by manipulating solute and solvent diffusion behaviors during the nanoprecipitation process. The findings reveal that solute diffusion at the liquid‐liquid interface of miscible solvent and antisolvent can be confined by self‐generated MQNs, which in turn dictates the morphology of the resulting nanoarchitectonics. By regulating interphase solute diffusion kinetics, a variety of MQN nanoarchitectonics are successfully produced with spherical, semifootball‐like, and bowl‐like morphologies, ranging in sizes from tens to hundred of nanometers. Notably, the non‐symmetric MQN nanobowls are further employed to create self‐propelled nanodrugs, which indicates enhanced efficacy in tumor therapy. This study underscores the critical role of liquid‐liquid interphase solute diffusion in determining the morphology and size of MQN nanoarchitectonics and highlights the potential of nanoprecipitation as a powerful technique for the precise fabrication of non‐symmetric nanoarchitectonics.

## Graphical abstract

Non‐symmetric metal‐quinone network (MQN) nanoarchitectonics with controllable morphology and size are constructed by manipulating interphase solute diffusion during the nanoprecipitation process. Self‐propelled nanomotors developed from non‐symmetric MQN nanoarchitectonics exhibit chemotactic autonomous motion toward overexpressed H_2_O_2_ in tumor microenvironment, which improves the antitumor efficacy due to the enhanced tumor penetration and cellular uptake.

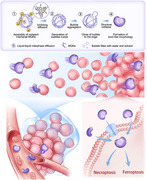

## 1 Introduction

Non‐symmetric nanoarchitectonics, characterized by structural heterogeneity and morphological non‐symmetry,^[^ ^1^ ^]^ offer several advantages over their symmetric counterparts, such as higher specific surface area,^[^ ^2^ ^]^ more reactive sites,^[^ ^3^ ^]^ and greater loading space.^[^ ^4^ ^]^ These features make them highly promising for a wide range of applications, including efficient energy storage,^[^ ^5^ ^]^ enhanced catalysts,^[^ ^6^ ^]^ and advanced nanomedicine.^[^ ^7^ ^]^ Specifically, non‐symmetric nanoarchitectonics hold significant potential for improving nanodrug delivery systems by facilitating biomolecule loading and release,^[^ ^8^ ^]^ enabling nanomotorized chemotactic motion,^[^ ^9^ ^]^ and enhancing nidus penetration and cellular uptake.^[^ ^10^ ^]^ To realize these benefits, various strategies have been developed to produce non‐symmetric nanoarchitectonics with flexible morphologies and versatile functions, including self‐sacrificing template,^[^ ^11^ ^]^ soft template,^[^ ^12^ ^]^ and emulsion polymerization.^[^ ^13^ ^]^ However, the use of templates or excipients in these methods raise concerns regarding drug loading efficiency, production cost, and, most importantly, biosafety. Thus, developing template‐ and surfactant‐free approaches is crucial to advance the biomedical applications of non‐symmetric nanoarchitectonics from concept to practical implementation.

Nanoprecipitation is a widely used method for preparing nanoparticles (NPs) by leveraging the diffusion of solute and solvent/antisolvent.^[^ ^14^ ^]^ During this process, interphase diffusion of solute and solvent can lead to three potential compositional states: (i) a uniform monophase domain with no NP formation, (ii) the Ouzo region, characterized by spontaneous solute nanoprecipitation, where interphase solute diffusion can be kinetically described by the Marangoni effect, driven by gradients in interfacial tension,^[^ ^15^ ^]^ and (iii) a solid/liquid diphase domain resulting from the complete interfacial breakdown, leading to uncontrolled interphase diffusion and solute precipitation.^[^ ^16^ ^]^ Despite its versatility, the application of nanoprecipitation for producing non‐symmetric nanoarchitectonics is rarely reported. A recent study demonstrated the formation of non‐symmetric nanoarchitectonics via polymer solidification at the liquid‐liquid interface, followed by restricted interphase diffusion.^[^ ^17^ ^]^ In this context, the localized assembly of amphiphilic homopolymers reinforced the interface, facilitating control over diffusion kinetics.^[^ ^18^ ^]^ However, stabilizing the liquid‐liquid interface in solvent/antisolvent system remains challenging, especially for hydrophobic small molecules with simple structures and weak intermolecular interactions.^[^ ^19^ ^]^ The introduction of additional building blocks capable of spontaneously assembling with small molecules may stabilize the interface via localized assembly and enable the regulation of interphase diffusion both within the Ouzo region and the diphase domain. It is well known that small molecules can coordinate with metal ions strongly and form robust metal ion‐small molecule networks.^[^ ^20^ ^]^ These coordination networks therefore offer significant potential to control liquid‐liquid interphase solute diffusion, promisingly expanding the scope of non‐symmetric nanoarchitectonics from polymers to small molecular drugs.

**Scheme 1:**
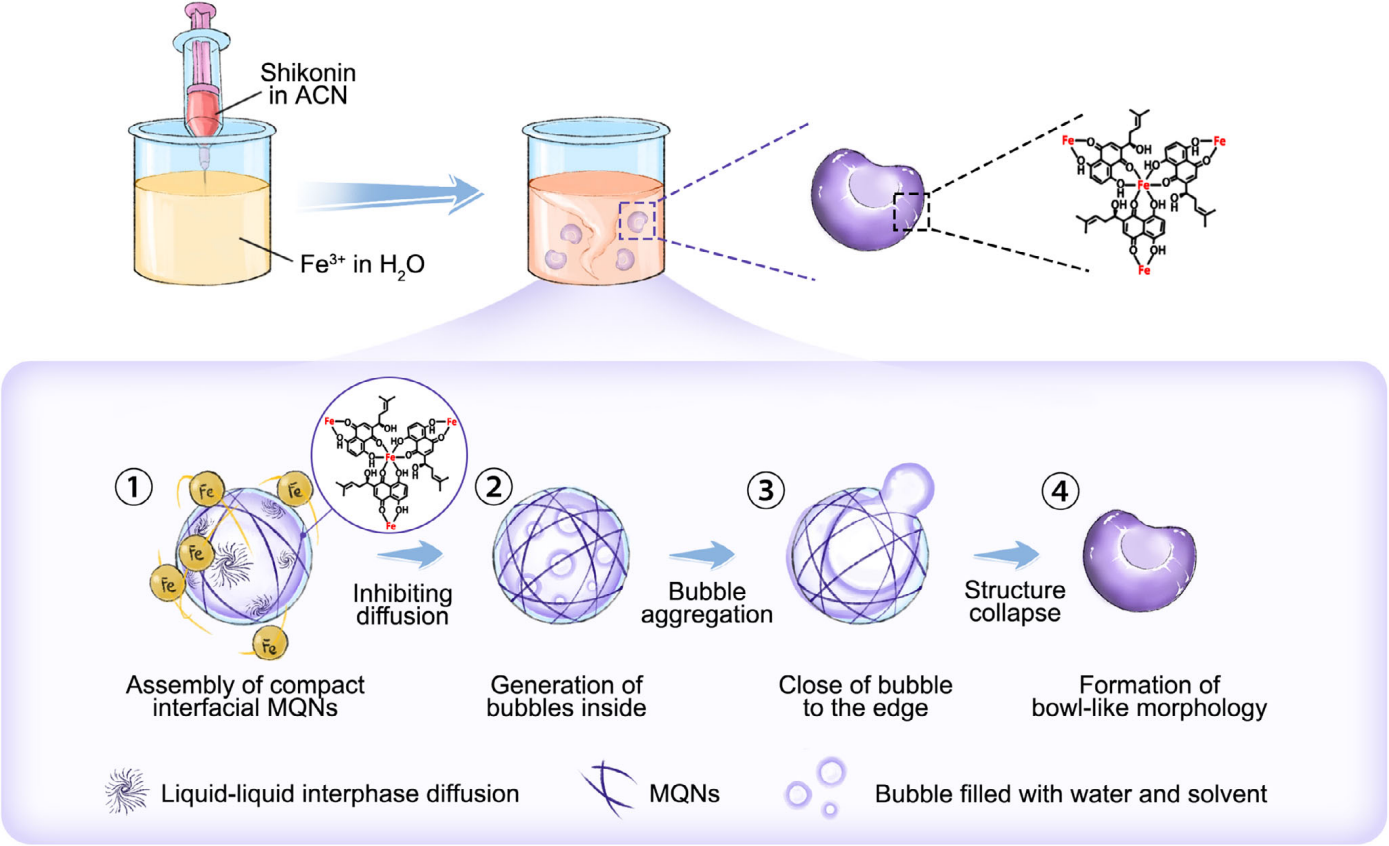
Schematic diagram of the preparation process (upper panel) and formation mechanism (lower panel) of non‐symmetric MQN nanoarchitectonics. The coordination‐driven assembly of Fe^3+^ and shikonin in ACN/water system results in non‐symmetric Fe/shikonin nanobowls. First, the interphase solute diffusion induces the assembly of compact MQNs at liquid‐liquid interface, which in turn inhibits solute diffusion. Then, bubbles filled with water and solvent generate and aggregate into a larger one inside interface. Next, larger bubbles close to the edge of interface. Finally, the nanostructure collapses, forming bowl‐like morphology.

Herein, we demonstrate the preparation of non‐symmetric metal‐quinone network (MQN) nanoarchitectonics by controlling interphase solute diffusion during nanoprecipitation (**Scheme** **1**). Upon leveraging self‐generated MQNs at liquid‐liquid interface between solvent and antisolvent, we precisely manipulate the diffusion of naphthoquinone across the interface. As a result, a variety of MQN nanoarchitectonics with different morphologies, including spherical, semifootball‐like, and bowl‐like shapes, are successfully formed. The non‐symmetric nanobowls are further engineered into self‐propelled nanomotors through enzyme modification, thereby enhancing their antitumor efficacy by promoting chemotactic tumor penetration. This work highlights the significance of precisely controlling solute diffusion at the liquid‐liquid interface, which plays a crucial role in determining the morphology and size of drug molecule assemblies during the nanoprecipitation process.

## 2 Results and Discussion

**Figure 1:**
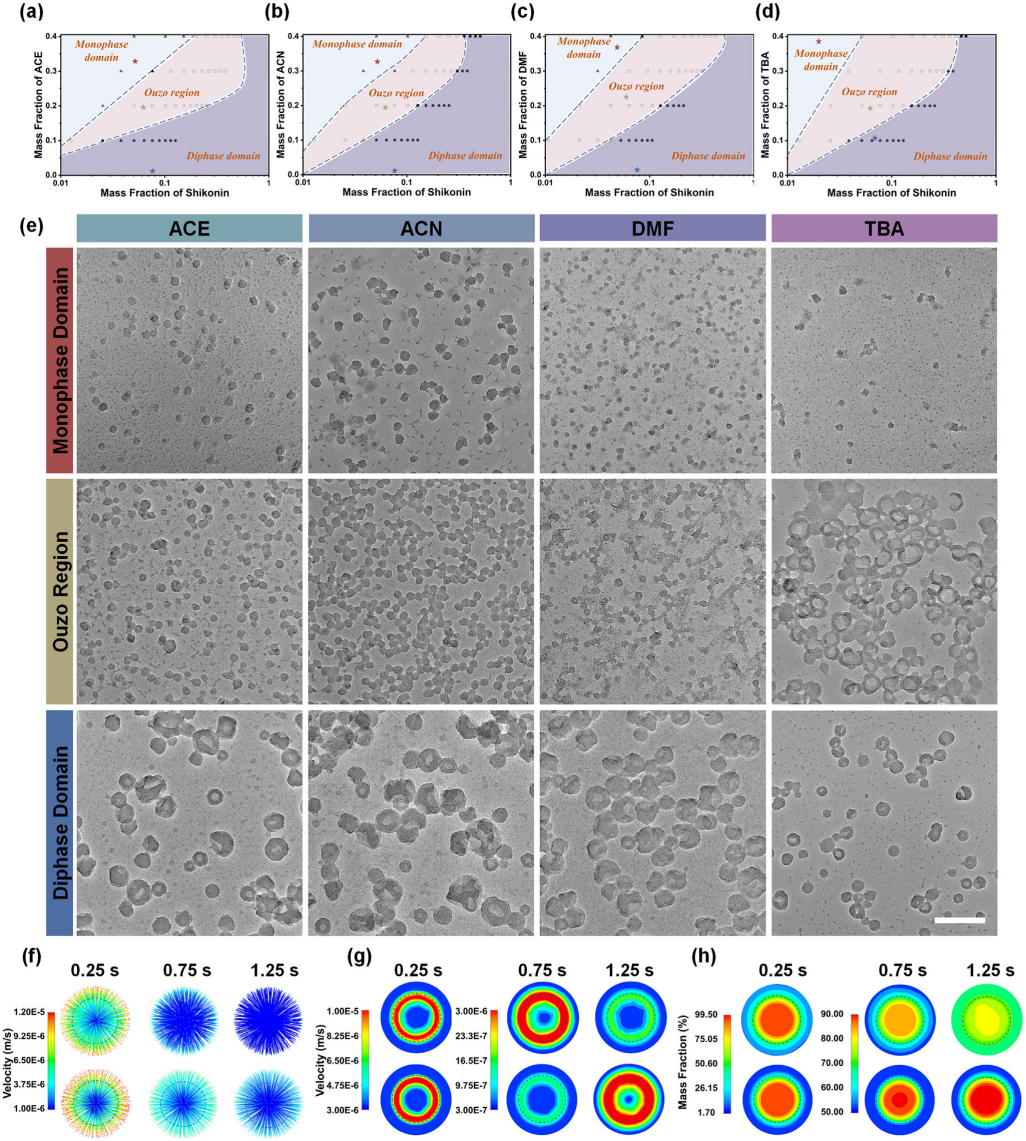
Phase diagrams of ACE/shikonin/water a), ACN/shikonin/water b), DMF/shikonin/water c), and TBA/shikonin/water d). e) TEM images of Fe/shikonin nanoarchitectonics prepared in monophase domain, Ouzo region, and diphase domain according to (a–d). The scale bar is 200 nm. CFD‐simulated velocity streamline figure f), total velocity contour plot g), and mass fraction of solvent h) in ACN/water (upper) and TBA/water (lower) system. Solvent droplet and water are inside and outside the black dashed line, respectively.

Shikonin, a representative water‐insoluble naphthoquinone drug featuring with π‐plane and quinonyl/phenolic hydroxyl that enable π‐π stacking and metal ion coordination, respectively,^[^ ^21^ ^]^ is chosen as a model compound to investigate interphase solute diffusion and interfacial MQN formation. Acetone (ACE), acetonitrile (ACN), *N*,*N*‐dimethylformamide (DMF), and *tert*‐butanol (TBA), each with different interfacial tension and permittivity, are used as solvents of shikonin to construct a variety of solvent/solute/antisolvent systems (Table S1, Supporting Information). **Figure** **1**a–d show the corresponding ternary phase diagrams derived from the experimental results, which is determined by both the solvent/water volume ratio and the final shikonin concentration. Water‐soluble Fe^3+^ is then introduced to coordinate with shikonin, forming Fe/shikonin networks in the monophase, Ouzo, and diphase domains. Transmission electron microscope (TEM) images reveal a spherical morphology of Fe/shikonin nanoarchitectonics in the monophase domain, independent of the solvents (Figure 1e). These spherical nanoarchitectonics likely result from a uniform distribution of shikonin and Fe^3+^ in the solvent/water phase, followed by a classical nucleation‐growth process of nanoprecipitation.^[^ ^22^ ^]^ In the Ouzo and diphase domains, solvent‐dependent morphologies of Fe/shikonin nanoarchitectonics are observed, including sphere, semifootball featured with multiple irregular holes on the surfaces, and bowl featured with open‐through structure with one opening hole on the surface. The sizes can be regulated from 20.1 ± 3.7 to 93.7 ± 17.3 nm under different conditions (Figure S1, Supporting Information). The variation in the size mainly results from the confined solute diffusion by the micro‐phase droplet with different sizes, which is governed by the composition of solvent/solute/antisolvent system related to solvent properties and solvent ratios. Regarding solvent properties, factors such as viscosity, relative permittivity and tension of solvents collectively influence the size of micro‐phase droplets and solute diffusion kinetics. Solvents with lower viscosity, lower relative permittivity, and higher interfacial tension tend to generate smaller micro‐phase droplets. These smaller droplets impose stronger confinement on solute diffusion, thereby leading to the formation of Fe/shikonin nanoarchitectures with reduced particle size. As for solvent ratios, increasing solvent‐to‐water ratio leads to smaller and more homogeneous micro‐phase droplets, which reduces the solute concentration per droplet. This, in turn, limits intra‐droplet solute diffusion and constrains the growth of nanostructures. As a result, regulating phase domain from diphase to Ouzo and monophase by controlling solvent composition facilitates the formation of Fe/shikonin nanoarchitectonics with smaller size. It is important to emphasize that these size‐regulating effects are not governed by a single factor but instead arise from the synergistic interplay between solvent properties and solvent ratios. Cryo‐TEM images further confirm that the non‐symmetric morphologies form in situ rather than during the drying process (Figure S2, Supporting Information). In contrast, micro‐ribbons rather than non‐symmetric nanoarchitectonics form in the absence of Fe^3+^ (Figure S3, Supporting Information). These findings suggest that small molecules can assemble into non‐symmetric nanoarchitectonics when metal ion coordination is introduced.

To reveal the effect of interphase solute diffusion in Ouzo region on the formation of non‐symmetric nanoarchitectonics, Marangoni effect is concerned (Figure S4, Supporting Information). When an TBA precursor solution containing both Fe^3+^ and shikonin is added into water, the resulting nanoarchitectonics present a spherical morphology. This spherical shape is attributed to the pre‐coordination of Fe^3+^ and shikonin, which likely drives a diffusion‐mediated nucleation‐growth process. In comparison, when shikonin TBA solution is added into Fe^3+^ aqueous solution, Marangoni effect‐driven interphase diffusion of both Fe^3+^ and shikonin occurs even without stirring. This process leads to the formation of compact Fe/shikonin networks at TBA/water interface, which impedes further solute diffusion across the interface and ultimately gives rise to non‐symmetric nanoarchitectonics. In addition, the partial suppression of Marangoni effect by diminishing the interfacial tension gradient using sodium dodecyl sulfate (SDS) weakens interphase solute diffusion and provides sufficient time for Fe^3+^‐shikonin coordination at the interface, thus promoting the formation of non‐symmetric nanoarchitectonics.

**Figure 2:**
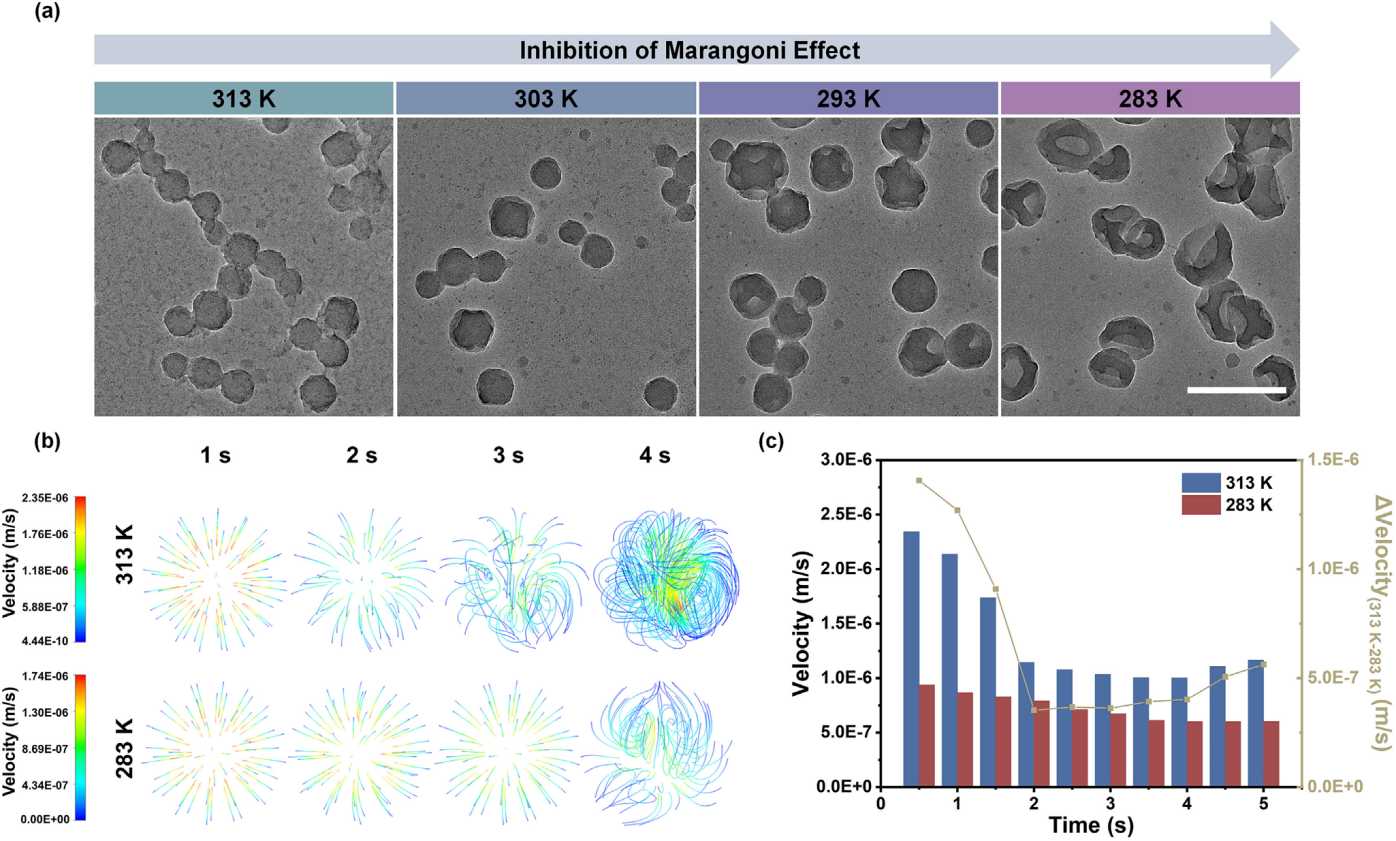
a) TEM images of Fe/shikonin nanoarchitectonics prepared at 313, 303, 293, and 283 K in Ouzo region. The scale bar is 200 nm. b) CFD‐simulated velocity streamline figure of ACN/water system at 313 and 283 K. c) The variation of CFD‐simulated velocity versus time at 313 and 283 K.

Computational fluid dynamics (CFD) simulations are further performed to investigate the solvent‐related Marangoni effect. As shown in Figure 1f,g, diffusion and total velocity are initially high in both ACN/water and TBA/water systems at 0.25 s. Between 0.25 and 1.25 s, diffusion and total velocity in the ACN/water system rapidly decline, with the ACN mass fraction equilibrium at ~70% (Figure 1h). This indicates that Marangoni effect intensely promotes interphase diffusion related to the superior miscibility of ACN with water, which in turn precludes the formation of Fe/shikonin networks at liquid‐liquid interface, resulting in spherical nanoarchitectonics (Figure 1e). For TBA/water system with poorer miscibility but greater interfacial tension gradient, the stronger interfacial tension‐related Marangoni effect counteracts the poor miscibility with water, which strengthens interphase diffusion and promotes interfacial MQN formation, leading to non‐symmetric nanoarchitectonics (Figure 1e). This means that interfacial tension‐related Marangoni effect can significantly influence interphase solute diffusion in Ouzo region. Additionally, a decrease in temperature reduces tension gradient, thereby weakening the Marangoni effect (Equation S1 in Supporting Information). As a result, the non‐symmetry of Fe/shikonin nanoarchitectonics increases as the temperature decreases from 313 to 283 K (**Figure** **2**a). CFD simulation reveals a diminished Marangoni effect and slower diffusion velocity at 283 K compared to 313 K (Figure 2b,c). The aforementioned results underscore the significant influence of Marangoni effect in the Ouzo region in determining NP morphology by driving interphase solute diffusion and inducing MQN formation at liquid‐liquid interface. However, an excessively strong Marangoni effect is disadvantageous to the formation of compact MQNs. Appropriate inhibition of Marangoni effect is necessary. The subsequent restriction of solute diffusion by the compact MQNs further causes the generation of bubbles containing water and solvent, followed by merging into a larger one inside interface.^[^ ^17^ ^]^ The larger bubble moves to the edge, which ultimately produces non‐symmetric nanoarchitectonics via the collapsing of nanostructures (Scheme 1).

**Figure 3:**
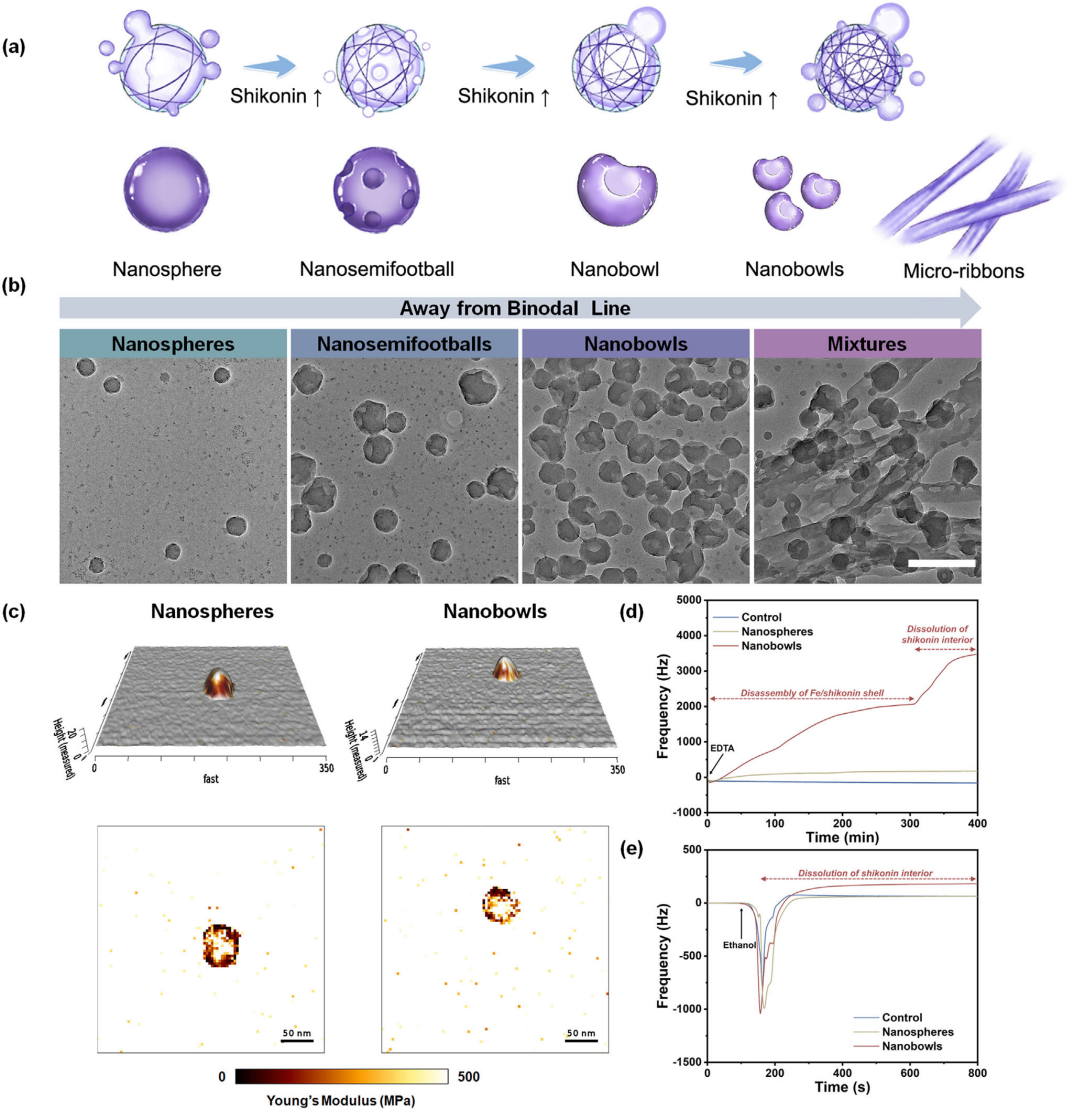
a) Schematic diagram of the morphological evolution of Fe/shikonin nanoarchitectonics as shikonin increases. b) TEM images of Fe/shikonin nanospheres, nanosemifootballs, and nanobowls, and the mixtures of Fe/shikonin nanobowls and shikonin micro‐ribbons. The scale bar is 200 nm. c) Representative 3D and 2D images of Young's modulus of Fe/shikonin nanospheres and nanobowls obtained by AFM. Frequency change from QCM monitoring of Fe/shikonin nanospheres and nanobowls upon the introduction of EDTA d) and ethanol e).

**Figure 4:**
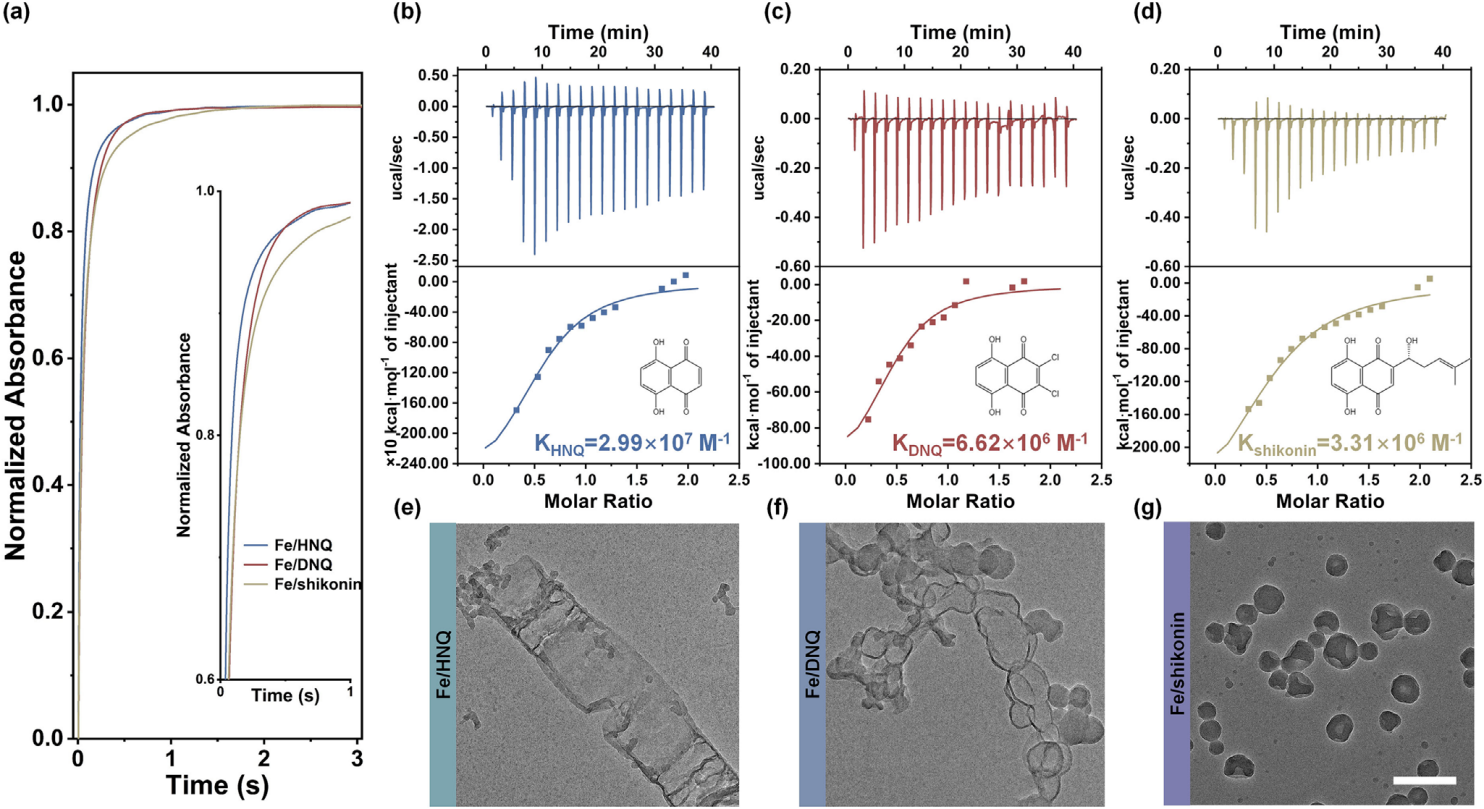
a) Kinetic curves of coordination between Fe^3+^ and naphthoquinones in ethanol obtained by a stopped‐flow experiment. ITC analysis of binding affinity between Fe^3+^ and HNQ b), Fe^3+^ and DNQ c), and Fe^3+^ and shikonin d). Insets: the structural formula of HNQ, DNQ and shikonin. TEM images of Fe/HNQ e), Fe/DNQ f) and Fe/shikonin g) nanoarchitectonics obtained in diphase domain. The scale bar is 200 nm.

The compact MQNs at liquid‐liquid interface also determine the morphology of nanoarchitectonics in diphase domain. As illustrated in **Figure** **3**a, increasing the concentration of shikonin beyond the binodal limitation facilitates the formation of Fe/shikonin networks by affecting the stoichiometry, which further restricts shikonin diffusion across the interface and promotes the formation of non‐symmetric nanoarchitectonics. Consequently, the morphology transforms from sphere to semifootball and eventually bowl as the increment of shikonin concentration (Figure 3b). Increasing shikonin‐to‐Fe^3^⁺ molar feed ratio from 1:2 to 3:2 results in a decrease in nanosemifootballs from 88% to 47%, while the proportion of nanobowls increases from 12% to 53%, which indicates the enhanced non‐symmetry (Figure S5, Supporting Information). However, excessive shikonin will disrupt the MQNs at liquid‐liquid interface, causing precipitation into micro‐ribbons (Figure 3b). Notably, the suppressive effect of the networks on solute diffusion is closely related to the coordination ability between naphthoquinone molecules and metal ions. To reveal this relationship, 5,8‐dihydroxy‐1,4‐naphthoquinone (HNQ) and 2,3‐dichloro‐5,8‐dihydroxy‐1,4‐naphthoquinone (DNQ), which exhibit distinct coordination behaviors with Fe^3+^, are studied. Stopped‐flow experiments show that both HNQ and DNQ coordinate with Fe^3+^ more rapidly than shikonin (**Figure** **4**a). Isothermal titration calorimetry (ITC) analysis shows that the association constants (*K*) of HNQ, DNQ, and shikonin with Fe^3+^ are 2.99 × 10^7^, 6.62 × 10^6^, and 3.31 × 10^6^ M^−1^, respectively (Figure 4b–d). Accordingly, the fastest coordination kinetics and largest coordination constant enable HNQ to easily cross the liquid‐liquid interface and coordinate with Fe^3+^, yielding Fe/HNQ nanospheres along with an abundance of HNQ micro‐ribbons (Figure 4e). An increase in the HNQ‐to‐Fe^3+^ molar feed ratio yields less Fe/HNQ nanospheres but more HNQ micro‐ribbons, owing to enhanced interface‐crossing and coordination due to HNQ's higher coordination affinity (Figure S6, Supporting Information). In contrast, DNQ, which has a weaker coordination ability with Fe^3+^, tends to form less compact MQNs at liquid‐liquid interface. This mildly restricts DNQ diffusion, leading to both solid and hollow Fe/DNQ nanospheres (Figure 4f). Correspondingly, fewer solid but more hollow Fe/DNQ nanospheres are formed at higher DNQ‐to‐Fe^3+^ molar feed ratio, due to restricted diffusion into water (Figure S7, Supporting Information). Because of the slowest coordination kinetics and smallest coordination constant, shikonin can sufficiently coordinate with Fe^3+^ at liquid‐liquid interface. This facilitates the formation of compact MQNs, which strongly restricts shikonin diffusion and results in only Fe/shikonin nanobowls (Figure 4g). Macroscopic diffusion experiments further confirm that solute diffusion can be effectively manipulated by regulating MQN properties (Figure S8, Supporting Information). Consequently, the morphology of as‐prepared MQN nanoarchitectonics is controlled by selecting metal ions with different coordination affinities toward shikonin (Figure S9, Supporting Information).

Previous studies have demonstrated that the construction of Fe/shikonin nanoarchitectonics mainly involves coordination interaction between Fe^3+^ and shikonin, as well as π‐π interaction among shikonin molecules.^[^ ^23^ ^]^ As shown in Fourier transform infrared (FTIR) spectra, for both nanospheres and nanobowls, the stretching vibration peaks at 3250 and 1606 cm^−1^ assigned to HO─C and C═O of shikonin shift to ~3450 and ~1529 cm^−1^, respectively (Figure S10, Supporting Information). These shifts indicate the formation of Fe–O coordination bonds between Fe^3+^ and shikonin at these two sites, which confirms the coordination‐driven assembly in both nanospheres and nanobowls. The structural configuration of shikonin within Fe/shikonin nanobowls are further investigated. Ethylenediaminetetraacetic acid (EDTA) and dimethyl sulfoxide (DMSO) are employed as competitive reagents to disrupt coordination and π‐π interactions, respectively.^[^ ^24^ ^]^ Compared to nanospheres, EDTA exerts a much weaker disruptive effect on nanobowls than DMSO, indicating fewer coordination interaction with Fe^3+^ and greater π‐π interaction within the nanobowls (Figure S11, Supporting Information). Inductively coupled plasma atomic emission spectra show a slightly lower Fe molar content (26.5%) in nanobowls compared to nanospheres (27.5%), consistent with fewer Fe^3+^‐shikonin coordination in nanobowls (Table S2, Supporting Information). These results suggest a gradient structure of nanobowls with Fe/shikonin‐dominated shell and shikonin‐dominated interior, supporting the notion that the as‐formed Fe/shikonin networks at liquid‐liquid interface inhibit interphase solute diffusion. The pronounced π‐π stacking of shikonin molecules imparts nanobowls with a higher Young's modulus relative to nanospheres, indicating that the tightly stacked shikonin inside the nanobowls better resists atomic force microscope (AFM) tip pressure (Figure 3c). Quartz crystal micro‐gravimetry (QCM) is further used to confirm the gradient structure of nanobowls, where an increase in frequency reflects mass loss. Upon introducing EDTA, the frequency of nanospheres rise slightly and continuously, indicating the gradual breakdown of Fe/shikonin networks. In contrast, nanobowls exhibits two distinct stages of frequency change. Namely, an initial rise from 0 to 310 min corresponds to the disassembly of Fe/shikonin shell at the surface, followed by the release of interior shikonin once the Fe/shikonin restriction is lifted (Figure 3d). When treated with ethanol, a noticeable frequency increase is observed for nanobowls, but not for nanospheres, implying the dissolution of shikonin interior in nanobowls (Figure 3e). TEM images further confirm that nanospheres remain intact and spherical, while nanobowls exhibit a hollow and bowl‐shaped morphology after ethanol treatment (Figure S12, Supporting Information). Overall, these results indicate that interfacial Fe/shikonin networks inhibit the diffusion of shikonin, promote its solidification, and ultimately lead to the formation of non‐symmetric nanoarchitectonics.

**Figure 5:**
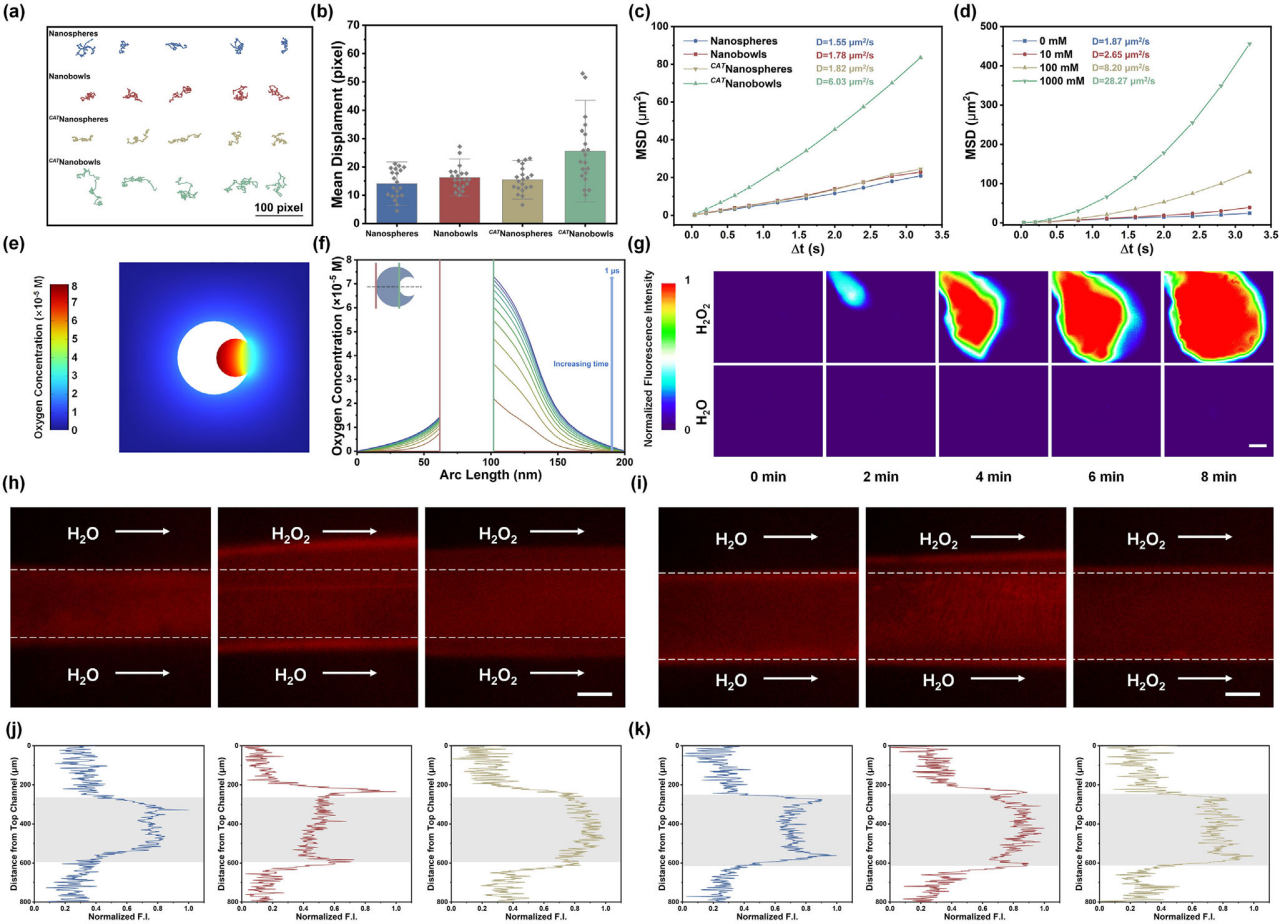
a) Representative tracking trajectories of nanospheres, nanobowls, *^CAT^* nanospheres, and *^CAT^* nanobowls in 100 mM H_2_O_2_ aqueous solution during 4 s. Mean displacement (n = 20) b) and MSD curves c) analyzed from tracking trajectories. Data are shown as mean ± SD and n represents the number of independent samples. d) MSD curves of *^CAT^* nanobowls in the presence of 0, 10, 100, and 1000 mM H_2_O_2_. e) Simulated concentration distribution of O_2_ in *^CAT^* nanobowls at 10 s. f) Simulated O_2_ concentration profile on the symmetric axis of *^CAT^* nanobowls from 0 to 1 µs with intervals of 0.1 µs. g) Normalized fluorescence quantitative images of *^CAT^* nanobowls in reservoir (ii) and (iii) of Y‐shaped device. The scale bar is 1000 µm. Representative fluorescence images h,i) and the corresponding fluorescent intensity distribution j,k) perpendicular to the direction of flow of *^CAT^* nanobowls (h,j) and *^CAT^* nanospheres (i,k) in the presence of different solution in the Ψ‐shaped microfluidic device. The white dashed line represents the edge boundary of laminar flow. The scale bar is 200 µm.

To enable the autonomous motion of nanobowls under tumor microenvironment (TME), catalase (CAT)‐loaded nanobowls (*^CAT^* nanobowls) are prepared as enzyme‐engineered nanomotors. *^CAT^* nanospheres are also prepared as an inactive control group. TEM‐mapping images show the presence of N element from CAT on the nanoarchitectonics (FigureS13a,b, Supporting Information). However, limited by the spatial resolution of EDS mapping, the distribution of N element at single‐particle level is unclear. Complementary negative‐staining TEM images reveal uniformly bright regions on the nanoarchitectonics surfaces, corresponding to the existing CAT (Figure S13c,d, Supporting Information). The observed nanoarchitectonics agglomeration in TEM images is primarily attributed to artifact introduced by negative staining and the drying process during sample preparation, rather than reflecting the native dispersion state in solution. This observation supports a random and uniform distribution of CAT on both spherical and bowl‐shaped nanoarchitectonics. The good colloidal stability of *^CAT^* nanospheres and *^CAT^* nanobowls in aqueous suspension is supported by dynamic light scattering (DLS) measurements, which shows nanometer‐sized hydrodynamic diameters and narrow polydispersity index (PDI) (Figure S13e,f, Supporting Information). The CAT loading capacity, determined by bicinchoninic acid assay protein assay kit, is 0.23 mg mg^−1^ of *^CAT^* nanobowls and 0.38 mg mg^−1^ of *^CAT^* nanospheres, respectively (Figure S14, Supporting Information). The O_2_ generation rates of equal amounts of *^CAT^* nanobowls and *^CAT^* nanospheres in 10 mM H_2_O_2_ are nearly identical, indicating that the enzyme activity contributes equally to the motion behaviors of nanoarchitectonics (Figure S15, Supporting Information). The ζ‐potential of Fe/shikonin nanobowls and CAT is +23.54 and ‐9.28 mV, respectively (Figure S16, Supporting Information). After combining, the ζ‐potential changes to +1.55 mV, which indicates that electrostatic attraction is the main driven force of enzymes adhering to Fe/shikonin nanoarchitectonics. The autonomous motion of *^CAT^* nanobowls is studied by tracking their trajectories using NP tracking analysis (NTA). As shown in **Figure** **5**a,b and Videos S1–S4 (Supporting Information), *^CAT^* nanobowls exhibit longer trajectories under H_2_O_2_ stimulus compared to *^CAT^* nanospheres, nanobowls, and nanospheres. The mean squared displacement (MSD) curve of *^CAT^* nanobowls displays a more parabolic shape and a higher diffusion coefficient (D = 6.03 µm^2^ s^−1^) compared to *^CAT^* nanospheres, which is attributed to the biocatalytic reaction on their non‐symmetric structure.^[^ ^25^ ^]^ In contrast, the MSD curves of nanobowls and nanospheres without CAT loading follow the linear Brownian motion pattern (Figure 5c).^[^ ^26^ ^]^ Furthermore, the diffusion coefficient D of *^CAT^* nanobowls increases from 1.87 to 2.65, 8.20, and 28.27 µm^2^ s^−1^ as the concentration of H_2_O_2_ increases from 0 to 10, 100, and 1000 mM, respectively, indicating fuel‐dependent self‐propelled motion (Figure 5d; Videos S5–S8, Supporting Information). The motion stability of *^CAT^* nanobowls is investigated through quantitative tracking of motion trajectories over 60 min. However, due to the H_2_O_2_ exhaustion during the measurement, the diffusion coefficient D declines from 9.15 to 5.10 µm^2^ s^−1^ within the initial 30 min, and further decreases to 2.10 µm^2^ s^−1^ at 60 min (Figure S17a, Supporting Information). Correspondingly, the mean displacement is 29.24, 24.21 and 19.53 pixel at 0, 30 and 60 min, respectively (Figure S17b, Supporting Information). Notably, the structure and motion behavior of *^CAT^* nanobowls remain stable in cell medium, laying a foundation for further applications in physiological environments (Figure S18, Supporting Information). To elucidate the motion mechanism driven by CAT‐catalyzed H_2_O_2_ decomposition, COMSOL multiphysics is employed to simulate bowl‐shaped nanomotors based on a 2D model. Figure 5e,f show the O_2_ concentration distribution around *^CAT^* nanobowls over time. Due to the spatial confinement effect, O_2_ significantly accumulates within the cavity, while O_2_ generated outside *^CAT^* nanobowls rapidly diffuses away. This creates a non‐symmetric gradient in O_2_ concentration, driving the self‐diffusiophoretic propulsion of *^CAT^* nanobowls.^[^ ^25^ ^]^

Chemotaxis of nanomotors towards stimuli‐overexpressed nidus tissue is highly desirable for enhancing precise treatment.^[^ ^27^ ^]^ To evaluate the chemotactic behavior of *^CAT^* nanobowls, both a static Y‐shaped device and a dynamic Ψ‐shaped microfluidic device using H_2_O_2_ as a chemoattractant are employed. In the static Y‐shaped device, *^CAT^* nanobowls are placed in reservoir (i), while reservoir (ii) and (iii) contain agarose gel with H_2_O_2_ and H_2_O, respectively (Figure S19, Supporting Information). As shown in Figure 5g, the fluorescence intensity in reservoir (ii) rapidly increases, whereas no fluorescence signal is detected in reservoir (iii). In contrast, *^CAT^* nanospheres rarely diffuse into either the H_2_O_2_ or H_2_O terminal reservoirs (Figure S20, Supporting Information). In the dynamic Ψ‐shaped microfluidic device, H_2_O_2_/H_2_O flow through ambilateral channels, while *^CAT^* nanobowls are pumped into the central channel (Figure S21, Supporting Information). The fluorescence signal of the *^CAT^* nanobowl fluid preferentially shifts to the H_2_O_2_ side (Figure 5h,j). In contrast, *^CAT^* nanospheres do not exhibit significant chemotactic shift toward either the H_2_O_2_ or H_2_O side (Figure 5i,k). These results confirm the chemotactic behavior of non‐symmetric *^CAT^* nanobowls in sensing areas with elevated H_2_O_2_ concentration, which is advantageous for facilitating endocytosis and traversing biological barriers to reach tumor tissues.

**Figure 6:**
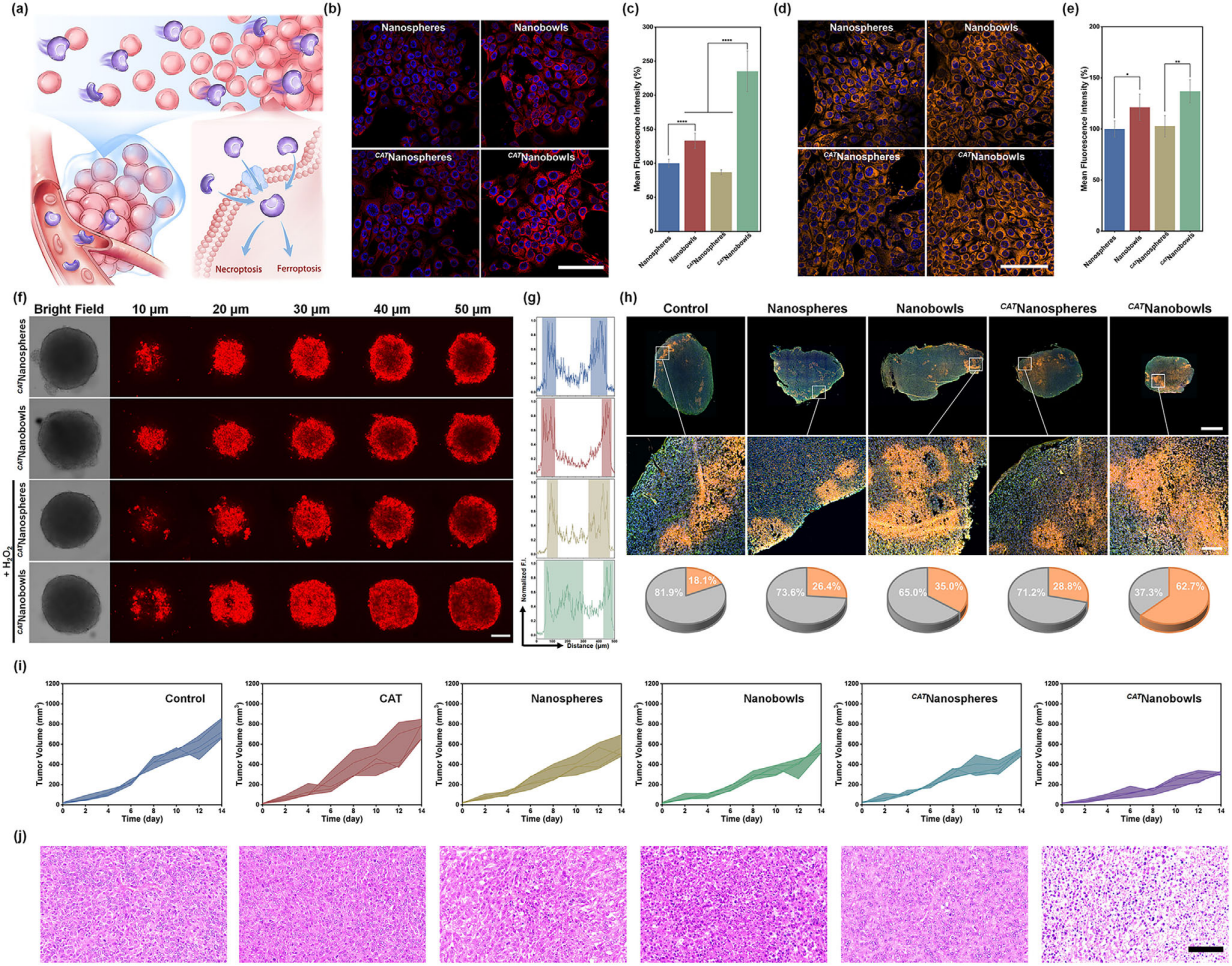
a) Schematic diagram of chemotactic *^CAT^* nanobowls for H_2_O_2_‐overexpressed tumor targeting, tumor penetration, and cell internalization. CLSM images b) and quantitative mean fluorescence intensity of red fluorescence (n = 3) c) of 4T1 cells after 6 h incubation with RhB‐labeled nanospheres, nanobowls, *^CAT^* nanospheres, and *^CAT^* nanobowls. The scale bar is 100 µm. CLSM images d) and quantitative mean fluorescence intensity of orange fluorescence (n = 3) e) of 4T1 cells after 6 h incubation with nanospheres, nanobowls, *^CAT^* nanospheres, and *^CAT^* nanobowls followed by staining with FerroOrange probe. The scale bar is 100 µm. The intensity of orange fluorescence reveals intracellular Fe^2+^ level. f) CLSM images for in‐depth scanning of 4T1 MTSs after incubation with RhB‐labeled *^CAT^* nanospheres and *^CAT^* nanobowls in the absence and presence of H_2_O_2_. The scale bar is 100 µm. g) Quantitative red fluorescence analysis of CLSM images on the 50 µm depth. h) CLSM images and corresponding quantitative orange fluorescence analysis of 4T1 tumor slice after different treatment for 24 h followed by staining with hoechst 33 342, actin‐tracker, and FerroOrange probes. The scale bars are 2000 (top) and 200 (bottom) µm. i) Individual tumor growth kinetics in different groups (n = 5). j) Representative H&E‐stained images of tumors collected from mice after different treatment for 14 days. The scale bar is 100 µm. Data are shown as mean ± SD and n represents the number of independent samples. * p < 0.05, ** p < 0.01, and **** p < 0.0001.

As shown in Figure S22a (Supporting Information), the cellular uptake of nanobowls by mouse breast carcinoma 4T1 cells is 1.76 times greater than that of nanospheres, attributed to their attachment to the cell membrane via high‐curvature edge.^[^ ^28^ ^]^ Detailed investigations reveal that the endocytic pathways for nanobowls involve caveolae‐mediated endocytosis, clathrin‐mediated endocytosis, and macropinocytosis simultaneously, whereas nanospheres are internalized solely through clathrin‐mediated endocytosis (Figure S22b, Supporting Information). This difference may be attributed to the influence of specific morphology on the interaction with cell membranes or proteins.^[^ ^29^ ^]^ The multi‐pathway endocytosis results in the increased cytotoxicity of nanobowls toward 4T1 cells compared to nanospheres (Figure S22c, Supporting Information). Upon loading CAT, the autonomous motion of *^CAT^* nanobowls further enhances tumor penetration and cellular uptake (**Figure** **6**a). As indicated in Figure 6b,c, confocal laser scanning microscopic (CLSM) images of 4T1 cells incubated with rhodamine B (RhB)‐labeled *^CAT^* nanobowls exhibit significantly greater fluorescence compared to the cells treated with *^CAT^* nanospheres, nanobowls, and nanospheres. Because the Fe^3+^‐shikonin coordination networks are disrupted in response to the overexpressed glutathione (GSH) under TME (Figure S23, Supporting Information),^[^ ^30^ ^]^ leading to the release of Fe^2+^ and shikonin to elicit ferroptosis and necroptosis,^[^ ^23^ ^]^ higher ferrous ion levels in *^CAT^* nanobowl‐treated cells is observed when stained with FerroOrange (Figure 6d,e). Consequently, *^CAT^* nanobowls exhibit greater cytotoxicity against 4T1 cells than against normal L929 cells (Figure S24, Supporting Information). In addition, enzyme‐linked immunosorbent assay (ELISA) test confirms the activation of both necroptosis and ferroptosis pathways in cells treated with nanospheres and nanobowls, independent of CAT loading, indicating that CAT has no negative impact on tumor treatment (Figure S25, Supporting Information). Furthermore, 3D multicellular tumor spheroids (MTSs) are cultured to investigate the capability of *^CAT^* nanobowls to penetrate biological barriers in vitro. As revealed by z‐stacking CLSM images, *^CAT^* nanobowls effectively penetrate into the MTSs, whereas other groups show significantly reduced infiltration within the dense cellular framework (Figure 6f,g; Figure S26, Supporting Information). These findings indicate that *^CAT^* nanobowls can achieve deep tissue penetration for effectively eradicating tumors.

The in vivo tumor penetration and efficacy of chemotactic *^CAT^* nanobowls are evaluated using an orthotopic 4T1 tumor model. Following intravenous injection of different formulations, the fluorescent area corresponding to Fe^2+^ within tumor tissue treated by *^CAT^* nanobowls reaches up to 62.7%, significantly higher than that of *^CAT^* nanosphere (28.8%), nanobowl (35.0%), nanosphere (26.4%), and the control group (18.1%) (Figure 6h). This indicates the effective tumor penetration and accumulation of *^CAT^* nanobowls. After treatment for 14 days, the tumor volume in the control and CAT groups increases to ~750 mm^3^, whereas it decreases to ∼550 mm^3^ in the groups treated with *^CAT^* nanospheres, nanobowls, and nanospheres (Figure 6i; Figure S27, Supporting Information). Notably, the treatment with *^CAT^* nanobowls reduces the tumor volume to ∼310 mm^3^. Hematoxylin‐eosin (H&E) staining of tumor tissues reveals more severe damage in the *^CAT^* nanobowl‐treated group compared to the other groups (Figure 6j). In addition, immunofluorescence staining of tumor tissues shows more pronounced ferroptosis and necroptosis in these tumors (Figure S28, Supporting Information). The aforementioned results verify that chemotactic *^CAT^* nanobowls enhance the efficacy of Fe/shikonin nanoarchitectonics through self‐propelled penetration and accumulation within tumors, effectively eradicating tumor cells via ferroptosis and necroptosis.

Finally, the biosafety of *^CAT^* nanobowls is evaluated. Throughout the treatment period, mice exhibit only slight fluctuations in body weight (Figure S29, Supporting Information). H&E staining of major organs reveal no observable damage (Figure S30, Supporting Information), and routine blood examinations detect no abnormalities (Figure S31, Supporting Information). These findings underscore the high biosafety of *^CAT^* nanobowls, ensuring their potential in nanotherapeutics.

## 3 Conclusion

In summary, a template‐ and surfactant‐free method is demonstrated for constructing non‐symmetric nanoarchitectonics from molecular drugs by employing MQNs to precisely control solute diffusion during the nanoprecipitation process. The regulation of the kinetics of interphase solute diffusion as well as the self‐generated MQNs at liquid‐liquid interface allows to achieve a variety of nanoarchitectonics morphologies including sphere, semifootball, and bowl. Nanomotors are further produced by engineering the nanobowls with CAT, which exhibit autonomous motion in response to H_2_O_2_. The H_2_O_2_ chemotaxis of nanomotors improves the uptake of tumor cells, thus enhancing the targeting and efficacy of drug nanoarchitectonics in treating tumor models. Our findings advance the fundamental understanding of nanoprecipitation technique in controlling the morphology of MQN nanoarchitectonics and extents the localized assembly strategy of small molecules to regulate interfacial diffusion kinetics. Building blocks such as polyphenols, photosensitizers, and flavonoids capable of localized assembly through robust intermolecular interactions represent promising candidates to construct state‐of‐the‐art non‐symmetric nanoarchitectonics. The concept of nano‐motorization highlights the potential for precisely fabricating non‐symmetric nanoarchitectonics composed entirely of drugs with the applications in self‐propelled nidus targeting.

## 4 Experimental Section

### Animal Experiments

All animal experiments were performed in accordance with the guidelines and regulations of Laboratory Animals of the First Hospital of Jilin University and approved by the Animal Laboratory Ethics Committee (No. JDYY20240496).

### Statistical Analysis

Raw data were not pre‐processed prior to analysis. All data with error bars were displayed as the mean ± standard deviation. Sample size (n) for each statistical analysis is in the relevant figure legends. Statistical significance was calculated by using two‐sided Student's t test, assigned at * p < 0.05, ** p < 0.01, *** p < 0.001 and **** p < 0.0001. Software used for statistical analysis is Origin 2021.

## Conflict of Interest

The authors declare no conflict of interest.

## Author Contributions

H.Z. supervised and proposed the project. W.Z. X., Y.L. and H.Z. designed the MQN nanoarchitectonics. W.Z. X. contributed to the preparation and characterization of MQN nanoarchitectonics, as well as wrote original draft. Y.K. X., Y.C., R.X.Y., and S.W.L. contributed to relevant characterization and assisted with part of cell experiments, bioimaging and animal model construction. Y.L. and H.Z. reviewed and edited the manuscript. All authors have given approvals to the final version of the manuscript.

## Supporting information



Supporting Information

Supplementary Video S1

Supplementary Video S2

Supplementary Video S3

Supplementary Video S4

Supplementary Video S5

Supplementary Video S6

Supplementary Video S7

Supplementary Video S8

## Data Availability

The data that support the findings of this study are available from the corresponding author upon reasonable request.
