# Supplementary material for: Manipulating Liquid‐Liquid Interphase Solute Diffusion for the Construction of Non‐Symmetric Metal‐Quinone Network Nanoarchitectonics and Nano‐Motorization
Source: Adv Sci (Weinh). 2025 Jul 3;12(37):e03164. doi: 10.1002/advs.202503164 (PMC12499380; doi:10.1002/advs.202503164)
Supplement: Supplementary file 1 — Supporting Information [file ADVS-12-e03164-s006.docx]

Supporting Information

Manipulating Liquid-Liquid Interphase Solute Diffusion for the Construction of Non-Symmetric Metal-Quinone Network Nanoarchitectonics and Nano-Motorization

Wenzhe Xu,^[a],[b]^ Yingke Xue,^[b]^ Yang Chen,^[b]^ Ruixu Yang,^[b]^ Shuwei Liu,^[c]^ Yi Liu,^[a],[b]^* and Hao Zhang^[a],[b],[c]^*

[a] W. Xu, Prof. Y. Liu, Prof. H. Zhang
Plastic and Reconstructive Surgery, The First Hospital of Jilin University, Changchun 130021, P. R. China
E-mail: yiliuchem@jlu.edu.cn; [hao_zhang@jlu.edu.cn](mailto:hao_zhang@jlu.edu.cn)

[b] W. Xu, Y. Xue, Y. Chen, R. Yang, Prof. Y. Liu, Prof. H. Zhang
State Key Laboratory of Supramolecular Structure and Materials, College of Chemistry, Jilin University, Changchun 130012, P. R. China

[c] Dr. S. Liu, Prof. H. Zhang
Joint Laboratory of Opto-Functional Theranostics in Medicine and Chemistry, Institute of Translational Medicine, The First Hospital of Jilin University, Changchun 130021, P. R. China

**Contents**

[**Experimental Section** 3](#_Toc196134263)

[**Materials** 3](#_Toc196134264)

[**Characterization** 3](#_Toc196134265)

[**Experimental Procedures and Computational Simulations** 3](#_Toc196134266)

[**Equation of Marangoni Effect** 8](#_Toc196134267)

[**Supplementary Figures and Tables** 9](#_Toc196134268)

[**Supplementary Movie List** 42](#_Toc196134269)

**Experimental Section**

**Materials**

Shikonin was purchased from Glip Biotechnology Co., Ltd. FeCl_3_∙6H_2_O, 5,8-dihydroxy-1,4-naphthoquinone (HNQ), 2,3-dichloro-5,8-dihydroxy-1,4-naphthoquinone (DNQ), tris(hydroxymethyl)aminomethane (Tris), ethylenediaminetetraacetic acid (EDTA), 2-deoxy-D-glucose (2-DG), chlorpromazine, methyl-β-cyclodextrin (M-βCD), simvastatin, nystatin, 5-(N-ethyl-N-isopropyl)-amiloride (EIPA), fluorescein isothiocyanate isomer I (FITC), catalase (CAT), agarose, rhodamine B (RhB) and *N*,*N*-dimethylformamide (DMF) were purchased from Aladdin. Acetone (ACE) was obtained from XiLong Scientific Co., Ltd. Acetonitrile (ACN) and *tert*-butanol (TBA) were purchased from Sinopharm Chemical Reagent Co., Ltd. Roswell Park Memorial Institute 1640 medium (RPMI) and Dulbecco’s modified Eagle’s medium (DMEM) were obtained from Gibco. Phosphate buffered saline (PBS) and penicillin/streptomycin solution were purchased from Biosharp. Fetal bovine serum (FBS) was purchased from Opcell. Hoechst 33342, actin tracker, bicinchoninic acid assay (BCA) protein assay kit and hydrogen peroxide assay kit were purchased from Beyotime. FerroOrange was purchased from Dojindo. Cell counting kit-8 (CCK-8) was purchased from GlpBio. Glutathione peroxidase 4 (GPX4) enzyme-linked immunosorbent assay (ELISA) assay kit was purchased from Jonlnbio. Receptor-interacting serine/threonine-protein kinase 3 (RIP3) and RIP1 ELISA assay kits were purchased from Spbio. GPX4 monoclonal antibody and RIP3 antibody were purchased from Proteintech. RIP1 antibody was obtained from Abmart.

**Characterization**

Ultraviolet-visible (UV-vis) absorption spectra were measured by using Shimadzu 2600 UV-vis spectrophotometer. Transmission electron microscopy (TEM) images were obtained by JEM-2100F electron microscope (Jeol) at 200 kV. The inductively coupled plasma atomic emission spectra (ICP-AES) were measured by using Agilent 725 (Agilent). Stopped-flow experiments were conducted by stopped flow spectrometer (Applied Photophysics SX20). Frequency shifts of nanoarchitectonics were monitored by Q-sense analyzer (Biolin). Isothermal titration calorimetry (ITC) analysis was conducted by using MicroCal iTC200 (Malvern). Young’s modulus was measured by atomic force microscope JPK Nanowizard 4xp (Bruker). Fourier transform infrared (FTIR) spectra were measured by a Bruker VERTEX 80V. Nanoparticle tracking analysis (NTA) of nanoarchitectonics was carried out by using Nanosight NS300 (Malvern). Fluorescent images of Y-shaped and Ψ-shaped devices were captured by BX53 biological microscope (Olympus). Flow cytometry was conducted by using Cytoflex (Beckman Coulter). Confocal laser scanning microscopic (CLSM) images were observed by using Nikon AX/AX R confocal microscope system (Nikon). Optical density of 96-well plates was measured by Infinite 200 Pro microplate reader (Tecan).

**Experimental Procedures and Computational Simulations**

**Preparation and manipulation of Fe/shikonin nanoarchitectonics in different compositional systems.**

In monophase domain, 2.0 mL shikonin solution (0.125 mg/mL in ACE, ACN, DMF or TBA) was added into 3.0 mL FeCl_3_∙6H_2_O aqueous solution (0.075 mg/mL). The reaction solution was centrifugated at 13500 rpm for 10 min and washed twice with deionized water to obtain spherical Fe/shikonin nanoarchitectonics.

In Ouzo region, 1.0 mL shikonin solution (0.25 mg/mL in ACE, CAN, DMF or TBA) was added into 3.0 mL FeCl_3_∙6H_2_O aqueous solution (0.075 mg/mL). The other procedures were the same as that in monophase domain. To evaluate the influence of temperature in Ouzo region, temperature of ACN solution was set as 283, 293, 303, and 313 K, and the reaction duration and purification process were fixed.

In diphase domain, 0.05 mL shikonin solution (5 mg/mL in ACE, ACN or DMF) was added into 3.0 mL FeCl_3_∙6H_2_O aqueous solution (0.075 mg/mL). As for TBA/shikonin/water system, 0.5 mL 0.05 mg/mL shikonin solution was used. The other procedures were the same as that in monophase domain. To control the interfacial Fe/shikonin networks, shikonin concentration in ACN was regulated as 1.25, 2.5, 7.5, and 10 mg/mL, and the reaction duration and purification process were fixed. To figure out the influence of coordination ability, HNQ and DNQ were employed instead of shikonin. To investigate the influence of stoichiometry, HNQ concentration in ACN was regulated as 0.79, 1.58, 4.75 and 6.33 mg/mL, and DNQ concentration in ACN was set as 1.08, 2.16, 6.48 and 8.63 mg/mL.

For further investigation, Fe/shikonin nanospheres were prepared by adding ACN solution containing 0.023 mL 5 mg/mL FeCl_3_∙6H_2_O and 0.05 mL 5 mg/mL shikonin into 3.0 mL H_2_O. The Fe/shikonin nanobowls were prepared according to the above-mentioned procedures in diphase of ACN/water system. To label FITC, 9.0 mL carbonate buffer solution (pH 8.0) containing 0.5 mg nanospheres or nanobowls was mixed with 0.02 mL 5 mg/mL FITC aqueous solution and reacted for 1 h. As for RhB-labeled nanoarchitectonics, 1.0 mL nanosphere or nanobowl aqueous solution at 0.5 mg/mL was mixed with 0.02 mL RhB aqueous solution at 1 mg/mL and stirred overnight.

**Computational fluid dynamics (CFD).**

The CFD was conducted by laminar flow module for simulating fluid dynamics in finite element analysis. The geometry model was set as two concentric spheres, where the diameters of two spheres were 10 and 15 μm. The whole area in 15 μm sphere was composed of water, except for the inside 10 μm sphere composed of solvent (i.e., ACN and TBA). The section corresponding to the maximal cross-sectional area of the concentric spheres was divided and smoothed into ~10^5^ unstructured grids with minimum mass and average mass of 0.2 and 0.9, respectively. This section was monitored for further investigation of mass fraction, total velocity, and diffusion velocity.

**Preparation of *^CAT^*nanospheres and *^CAT^*nanobowls.**

First, 0.1 mL CAT (20000 unit/mg protein) was added into 9.0 mL Tris-HCl buffer (pH 8.5) containing 0.5 mg nanospheres or nanobowls. After stirring for 8 h, *^CAT^*nanospheres or *^CAT^*nanobowls were obtained by centrifugation at 13500 rpm for 15 min and wash twice with deionized water. For RhB-labeled nanoarchitectonics,1.0 mL *^CAT^*nanosphere or *^CAT^*nanobowl aqueous solution at 0.5 mg/mL was mixed with 0.02 mL RhB aqueous solution at 1 mg/mL and stirred overnight.

**Analysis of movement measured by NTA.**

NTA was applied to investigate the movement of nanospheres, nanobowls, *^CAT^*nanospheres, and *^CAT^*nanobowls in H_2_O_2_. Briefly, 100 mM H_2_O_2_ at concentration of 10^7^~10^9^ particles/mL was used for NTA measurement. The record duration was 30 s. The NTA software was applied to track the trajectories of various nanoarchitectonics. To evaluate the fuel-dependent movement of *^CAT^*nanobowls, the concentration of H_2_O_2_ was set as 0, 10, 100, and 1000 mM. The mean squared displacement (MSD) of each particle was calculated as following equation according to the previous reports:^[1]^

$MSD\left( \Delta t \right)=\left\langle\left[ x_{i}\left( t+\Delta t \right)-x_{i}\left( t \right) \right]^{2} \right\rangle$ *i*=2 for 2D analysis

Then, the diffusion coefficient D was extracted as following equation:

$$MSD\left( \Delta t \right)=4D\Delta t$$

20 particles of each group were analyzed for average MSD.

**Computational simulations of O_2_ concentration distribution.**

The finite element analysis was used to simulate the O_2_ concentration distribution around *^CAT^*nanobowls in 1% H_2_O_2_. The simulations were conducted in a 2D mode. The catalytic reaction of H_2_O_2_ by *^CAT^*nanobowls was 0.024 min^-1^ according to Figure S15. The generation of O_2_ was indicated by the surface flux of 0.00143 mol m^−2^ s^−1^. Fick’s law was applied for simulations.

Calculation of the surface flux of O_2_:

The catalytic reaction rate ($r_{H_{2}O_{2}}$) of 3.0 mL 0.1% H_2_O_2_ solution in the presence of 1.0 μg *^CAT^*nanobowls is calculated as following:

$$r_{H_{2}O_{2}}=\frac{3.0 ml\times(0.1\%-0.0939\%)\times\rho_{H_{2}O_{2}}}{M_{H_{2}O_{2}}\times600}=9.97\times{10}^{-9} mol s^{-1}$$

where $\rho_{H_{2}O_{2}}$= 1.13 g/mL is the density of H_2_O_2_, and $M_{H_{2}O_{2}}$= 34 g/mol is the molar mass of H_2_O_2_.

The production rate of O_2_ ($r_{O_{2}}$) in 1.0 mL 0.1% H_2_O_2_ solution containing 1.0 μg *^CAT^*nanobowls is:

$$r_{O_{2}}=\frac{1}{2}\times r_{H_{2}O_{2}}=4.85\times{10}^{-9} mol s^{-1}$$

Due to the first-order reaction feature, the production rate of O_2_ ${(r}_{O_{2}}^{'})$is proportional to the concentration of H_2_O_2_ solution in the catalytic decomposition of H_2_O_2_.

$$r_{O_{2}}^{'}=10\times r_{O_{2}}=4.85\times{10}^{-8} mol s^{-1}$$

The surface flux of O_2_ ($f_{O_{2}}$) on *^CAT^*nanobowls is:

$$f_{O_{2}}=\frac{r_{O_{2}}^{'}}{S\times1.0 \mu g}=0.00143 mol m^{2}s^{-1}$$

Where $S=34.98 m^{2}g^{-1}$ is the specific surface area of *^CAT^*nanobowls obtained from measured mass and volume of *^CAT^*nanobowls.

**Evaluation of chemotaxis behaviors in static Y-shaped devices.**

Y-shaped device was established according to the previous report.^[2]^ Briefly, the length and width of main channel were 1 and 0.4 cm, respectively, and the branch channel possessed 0.7 cm length and 0.3 cm width. 5 M H_2_O_2_ aqueous solution and H_2_O were mixed with the equal volume of heated agarose solution (10 mg/mL) and then added into reservoir (ii) and (iii), respectively. After cooling to room temperature, 700 μL water was added into the Y-shaped device. Then, 30 μL RhB-labeled *^CAT^*nanospheres or *^CAT^*nanobowls were accurately dropped into the reservoir (i). The fluorescent images of reservoir (ii) and (iii) were recorded at specific time points by using fluorescence microscope. Fiji software was used for the image quantification and pseudo-color. The H_2_O_2_ concentration in different positions of Y-shaped device was detected by using hydrogen peroxide assay kit after filling the whole channels with water for 5 min.

**Evaluation of chemotaxis behaviors in dynamic** **Ψ-shaped microfluidic devices.**

Ψ-shaped device was established according to the previous report.^[2]^ The length, width and height of three-inlet one-outlet Ψ-shaped device were 2.2 cm, 1.5 mm and 300 μm, respectively. 10 mM H_2_O_2_ aqueous solution and H_2_O flowed in each side of the side channels, respectively. The RhB-labeled *^CAT^*nanospheres or *^CAT^*nanobowls flowed in the middle channel. The flow velocity of each channel was 0.01 mm/min. The fluorescent images of the main channel were recorded at specific time points by using fluorescence microscope. Fiji software was used for the image quantification.

**Cell culture.**

Mouse breast carcinoma 4T1 cells were cultured with complete medium composed of RPMI, 10% FBS, and 1% penicillin/streptomycin in the condition with 5% CO_2_ at 37 °C. As for mouse fibroblast L929 cells, DMEM was used instead of RPMI.

**Study of cellular uptake.**

To quantify the cellular uptake of nanospheres or nanobowls, 4T1 cells were collected after incubation with 100 μg/mL nanospheres or nanobowls for 6 h. Then, the 4T1 cells were divided into two parts for ICP-AES after ablation with aqua regia and for BCA protein assay, respectively.

To investigate the endocytosis pathway of nanospheres and nanobowls, 4T1 cells were pre-treated with different endocytosis inhibitors for 40 min, including 2-DG (60 mM), chlorpromazine (30 μM), M-βCD (5 mM), simvastatin (30 μM), nystatin (5 μM) and EIPA (50 μM). After that, the 4T1 cells were incubated with 50 μg/mL FITC-labeled nanospheres or nanobowls for 6 h. Eventually, the 4T1 cells were collected and resuspended in PBS for flow cytometry with excitation at 488 nm.

To investigate the cellular uptake of nanospheres, nanobowls, *^CAT^*nanospheres, and *^CAT^*nanobowls, 4T1 cells were seeded in 20 mm CLSM-exclusive culture dishes and attached overnight, and then co-cultured with 50 μg/mL various RhB-labeled nanoarchitectonics for 6 h. After removing medium and washed with PBS for thrice, the cells were stained with hoechst 33342 for 10 min. Finally, CLSM was used for capturing fluorescent images with excitation at 405 and 561 nm. Quantitative analysis of RhB fluorescence was carried out by ImageJ. For ferrous ion detection, 4T1 cells in 20 mm CLSM-exclusive culture were treated with 50 μg/mL nanospheres, nanobowls, *^CAT^*nanospheres, and *^CAT^*nanobowls for 6 h before stained with FerroOrange for 30 min. Quantitative analysis of FerroOrange fluorescence was carried out by ImageJ.

**Cytotoxicity assay.**

To evaluate the cytotoxicity of nanospheres and nanobowls, 4T1 cells seeded in 96-well plates were treated with specific concentration of nanospheres or nanobowls for 24 h. Then, the medium in each well was replaced with medium composed of 10% CCK-8. After incubating in dark at 37 °C for 40 min, the optical density at 450 nm was measured by using microplate reader to evaluate cell viability. With respect to the cytotoxicity of *^CAT^*nanobowls towards 4T1 and L929 cells, nanospheres or nanobowls were replaced with *^CAT^*nanobowls.

**Intracellular expressions of RIP1, RIP3, and GPX4.**

4T1 cells seeded in 6-well plates were treated with medium, 30 μg/mL nanospheres, nanobowls, *^CAT^*nanospheres, and *^CAT^*nanobowls for 24 h. After that, the cells were collected and lysed. Finally, the lysate was used for detection of RIP1, RIP3, and GPX4 by ELISA assay kits.

**Investigation of** **penetration ability.**

To establish 3D multicellular tumor spheroids (MTSs), 4T1 cells were seeded in U-shaped low-attachment 96-well plates for 2 days. Then, compact 3D MTSs with similar sizes were incubated with 20 μg/mL RhB-labeled *^CAT^*nanospheres and *^CAT^*nanobowls in the absence and presence of 5 mM H_2_O_2_ for 6 h. After that, the cells were carefully transferred into CLSM-exclusive culture dishes. Finally, the z-stacking fluorescent images were observed by CLSM. Quantitative analysis of RhB fluorescence was carried out by ImageJ.

**Animals.**

Female Balb/c mice (6 weeks, 18-20 g) were purchased from Beijing Vital River Laboratory Animal Technology Co., Ltd. All animal experiments were performed in accordance with the guidelines and regulations of Laboratory Animals of the First Hospital of Jilin University and approved by the Animal Laboratory Ethics Committee (No. JDYY20240496).

***In vivo* studies of penetration ability.**

To establish the orthotopic 4T1 breast tumor model, 1×10^6^ 4T1 cells were injected subcutaneously into the right mammary fat pads of Balb/c mice. When the tumor reached ~200 mm^3^, mice were injected intravenously with 10 mg/kg nanospheres, nanobowls, *^CAT^*nanospheres, and *^CAT^*nanobowls, except for the control group without treatment. After 24 h post injection, tumors were collected and sliced at the section corresponding to the maximal cross-sectional area of the tumor tissue. Then, the tumor tissue slices were stained with hoechst 33342, actin tracker and FerroOrange. Finally, the fluorescent images were observed by CLSM. Quantitative analysis of FerroOrange fluorescence was carried out by ImageJ.

***In vivo* anti-tumor effect.**

First, orthotopic 4T1 breast tumor model was established on female Balb/c mice. After 5 days, mice were randomly divided into six groups with five mice per group: control, CAT (at an equivalent dosage of 10 mg/kg *^CAT^*nanobowls), nanospheres (10 mg/kg), nanobowls (10 mg/kg), *^CAT^*nanospheres (10 mg/kg), and *^CAT^*nanobowls (10 mg/kg). The mice were intravenously injected with different formulations on the 0^th^ and 4^th^ day. The volume of tumor and the body weight of an individual mouse were recorded every other day up to 14 days. Tumor volume was calculated according to the formula: tumor volume = length × width × width × 0.5 mm^3^. After 14 days, mice were sacrificed to collect blood for blood routine examination. The major organs including heart, liver, spleen, lungs and kidneys were collected for hematoxylin and eosin (H&E) staining analysis. The tumors were collected for H&E staining analysis and immunofluorescence analysis of necroptosis and ferroptosis.

**Statistical analysis.**

Raw data were not pre-processed prior to analysis. All data with error bars are displayed as the mean ± standard deviation. Statistical significance was calculated by using two-sided Student’s t test, assigned at * p < 0.05, ** p < 0.01, *** p < 0.001 and **** p < 0.0001. Software used for statistical analysis is Origin 2021.

**Equation of Marangoni Effect**

According to the previous reports,^[3]^ Marangoni effect is summarized as following equation:

$M_{a}=\frac{\left| \Delta\gamma\right|\cdot l}{\mu\cdot D}$ Eq. S1

where *Δγ* represents the tension gradient between solvent and antisolvent at liquid-liquid interface, and $l$ is representative length (here, radius of a solvent droplet). 𝜇 is the dynamic viscosity of solvent, and *D* is the diffusion coefficient of solvent/antisolvent solution.

In mechanism, tension gradient plays a predominant role in manipulation of Marangoni effect in Ouzo region. Therefore, temperature is considered as a major factor to regulate tension gradient.^[4]^

**Supplementary Figures and Tables**

**Table S1.** The interfacial tension and relative permittivity of ACE, ACN, DMF, TBA, and H_2_O at 298 K.^[4]^

| Solvent | TBA | ACE | ACN | DMF | H_2_O |
| --- | --- | --- | --- | --- | --- |
| Tension (mN/m) | 20.01 | 22.71 | 28.66 | 35.74 | 72.06 |
| Relative permittivity | 12.34 | 20.49 | 35.69 | 37.22 | 78.36 |


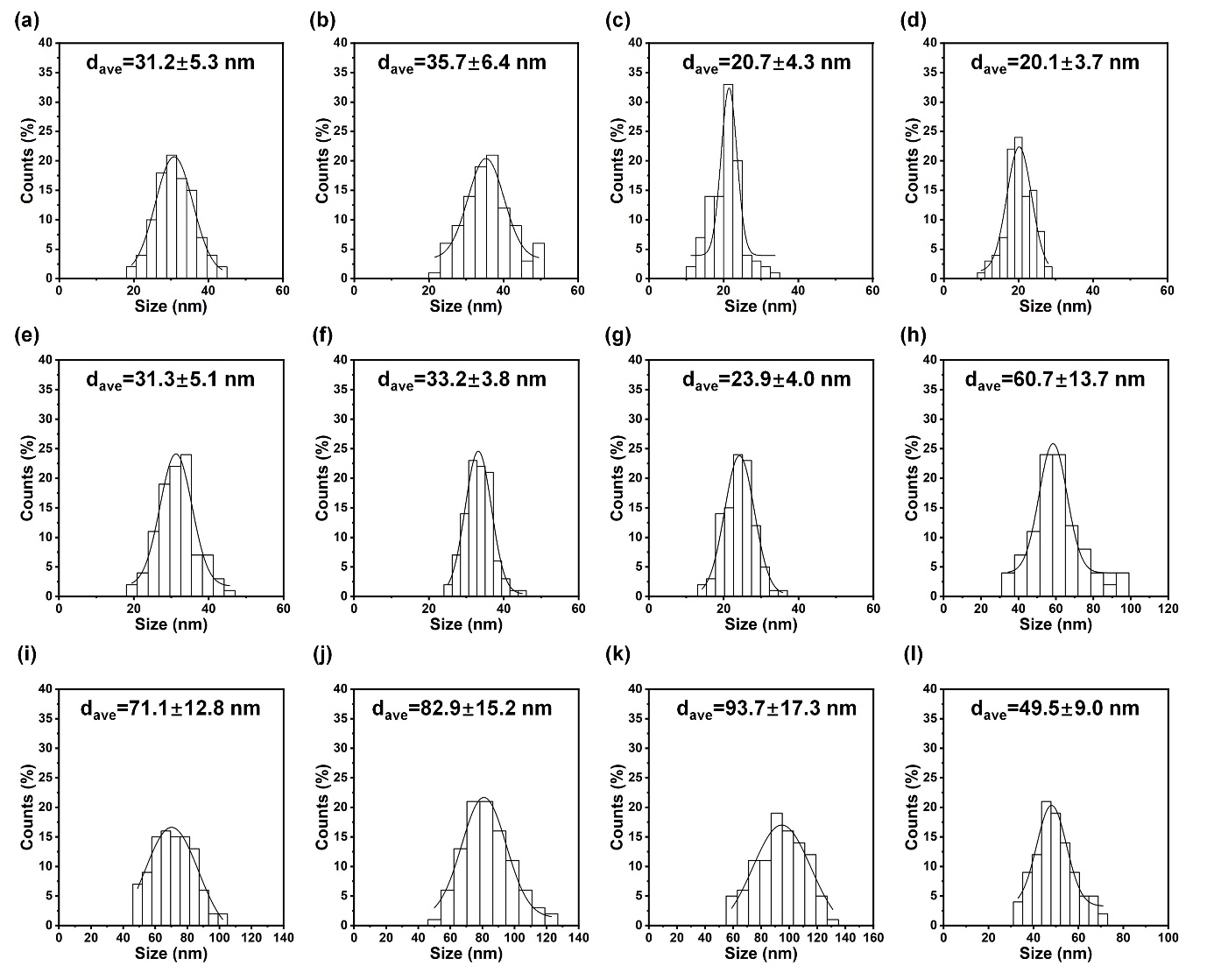


**Figure S1.** TEM size distributions of Fe/shikonin nanoarchitectonics prepared in monophase (a-d), Ouzo (e-h) and diphase (i-l) domain of ACE/shikonin/water (a, e and i), ACN/shikonin/water (b, f and j), DMF/shikonin/water (c, g and k), and TBA/shikonin/water (d, h and l). A minimum of 100 particles are analyzed per sample.


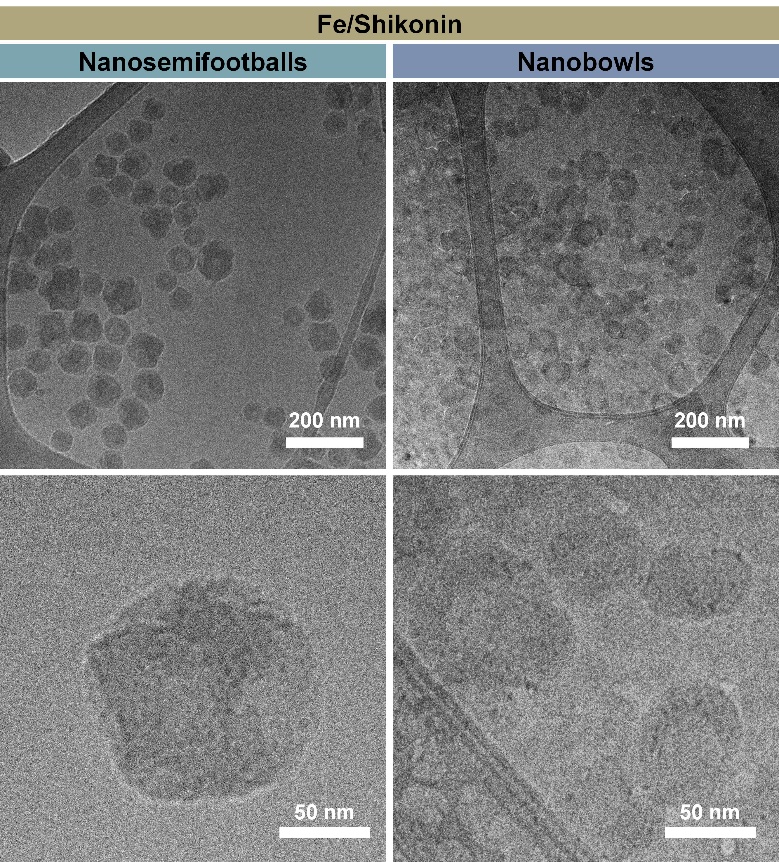


**Figure S2.** Cryo-TEM images of Fe/shikonin nanosemifootballs and nanobowls prepared with ACN and water.


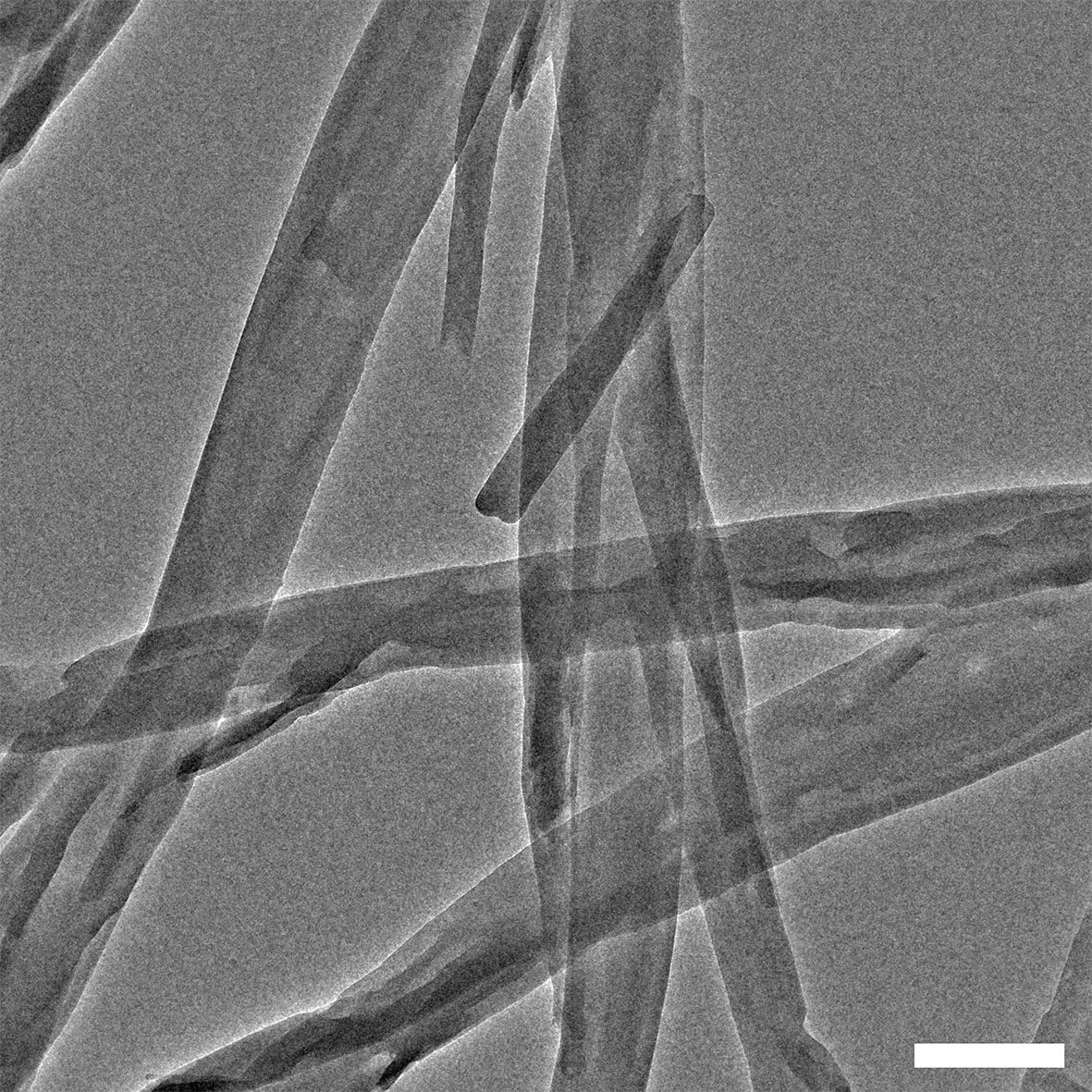


**Figure S3.** TEM image of shikonin micro-ribbons in water in the absence of Fe^3+^. The scale bar is 500 nm.


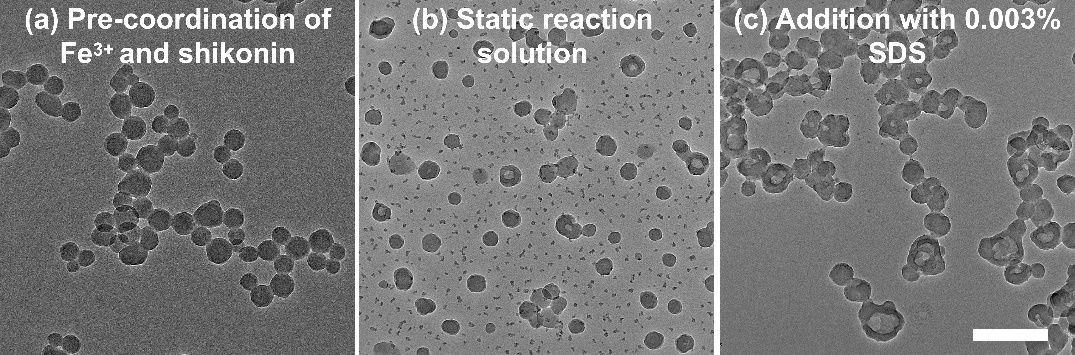


**Figure S4.** TEM images of Fe/shikonin nanoarchitectonics prepared with different procedures to demonstrate the spontaneous Marangoni effect at liquid-liquid interface. The scale bar is 200 nm. (a) TBA solution of both Fe^3+^ and shikonin with molar feed ratio of 1:1 is added into water. (b) Shikonin TBA solution is added into Fe^3+^ aqueous solution without stirring, and the reaction solution stands for 30 min. (c) On the basis of typical preparation procedure of non-symmetric Fe/shikonin nanoarchitectonics as mentioned in the Experiment Section, 0.003% SDS is added to reduce tension gradient.


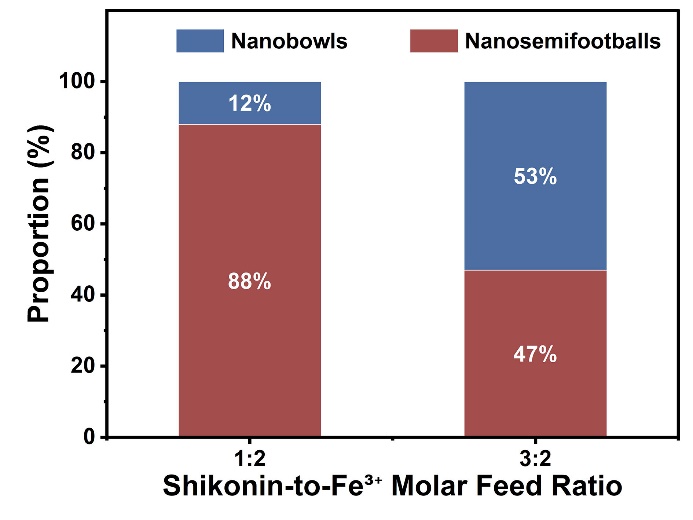


**Figure S5.** Proportion of nanobowls and nanosemifootballs in the products obtained at different shikonin-to-Fe³⁺ molar feed ratios.


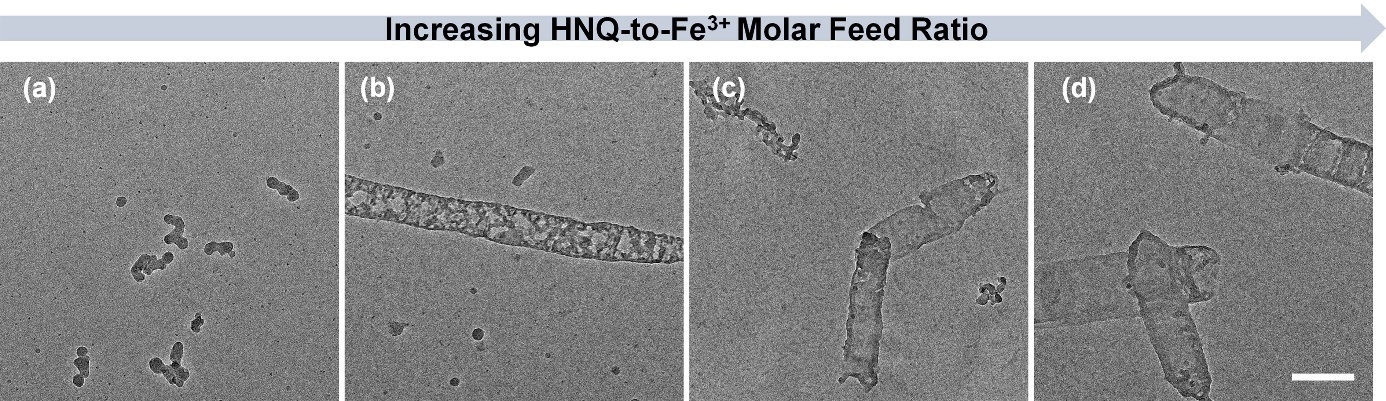


**Figure S6.** TEM images of Fe/HNQ nanoarchitectonics obtained in diphase domain with the increment of HNQ-to-Fe^3+^ molar feed ratios from 0.25:1 (a) to 0.5:1 (b), 1.5:1 (c), and 2:1 (d). The scale bar is 200 nm.


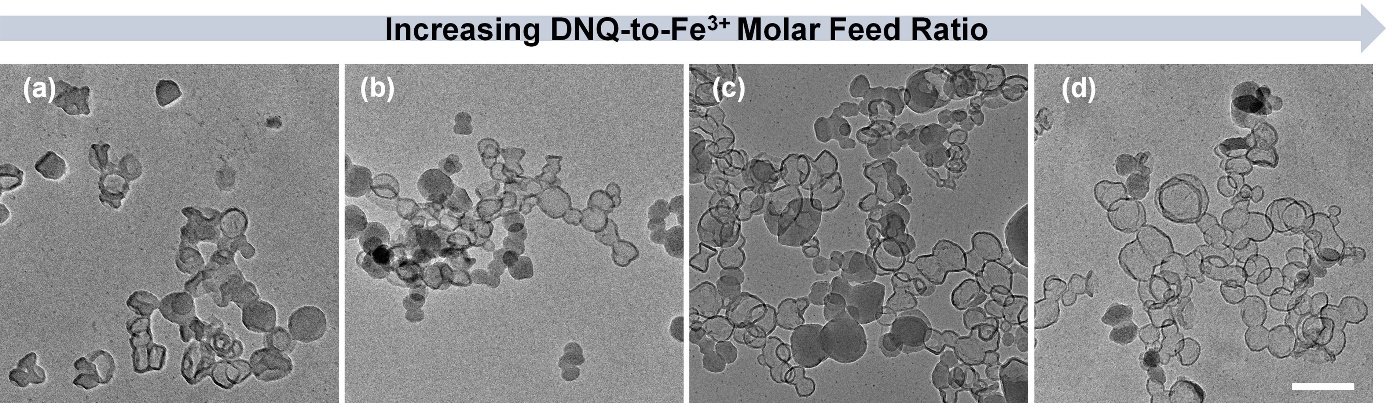


**Figure S7.** TEM images of Fe/DNQ nanoarchitectonics obtained in diphase domain with the increment of DNQ-to-Fe^3+^ molar feed ratios from 0.25:1 (a) to 0.5:1 (b), 1.5:1 (c), and 2:1 (d). The scale bar is 200 nm.

**
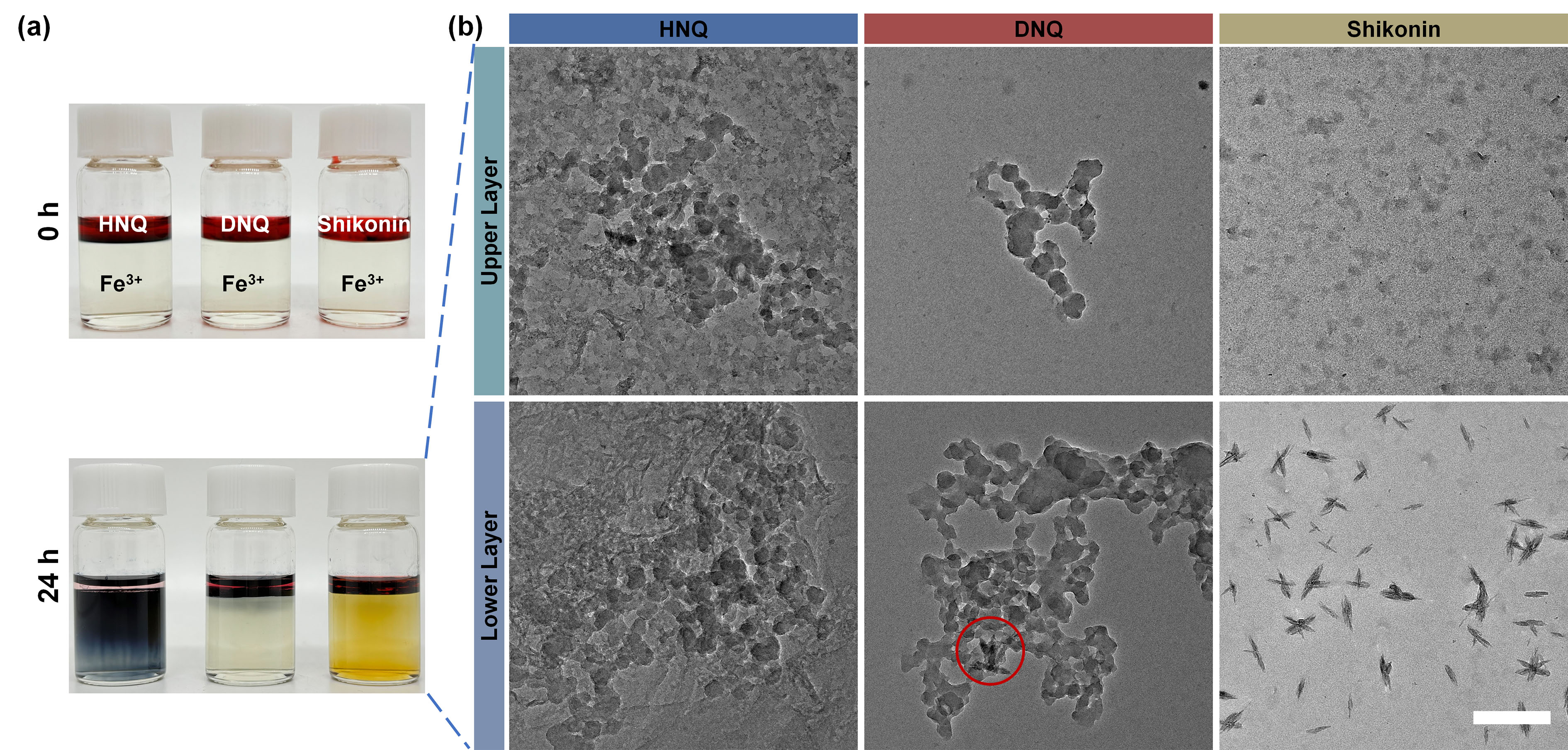
**

**Figure S8.** Macroscopic diffusion experiments for confirming naphthoquinone diffusion influenced by MQNs. (a) Optical photographs of macroscopic diffusion experiments. Water-immiscible *n*-Butanol solution containing HNQ, DNQ, or shikonin is on the upper layer, while Fe^3+^ aqueous solution is in the lower layer. At 0 h, black Fe^3+^-HNQ complex form rapidly, followed by Fe^3+^-DNQ. While the black Fe^3+^-shikonin complex is few. After 24 h, HNQ diffuses into water and coordinates with Fe^3+^ in the lower layer, leading to obvious black Fe^3+^-HNQ aqueous solution. A part of DNQ diffuses and coordinates, resulting in light black Fe^3+^-DNQ aqueous solution. Because of a few shikonin diffuses, Fe^3+^ hydrolysis occurs spontaneously in the lower layer, making aqueous solution yellow. (b) TEM images of product in upper and lower layers in different 24 h-reacted solution. The scale bar is 200 nm. Fe/HNQ and Fe/DNQ nanospheres are observed in both upper and lower layers. Note that few shuttle-like FeOOH particles are observed in lower layer for DNQ group (in red circle), indicating the hydrolysis of residual Fe^3+^.^[5]^ Fe/shikonin nanospheres and shuttle-like FeOOH particles are in upper and lower layers, respectively. These phenomena demonstrate faster coordination kinetics of HNQ and DNQ with Fe^3+^ compared to shikonin, consistent with stopped-flow and ITC results. As a result, HNQ and DNQ exhibit more favorable trends to diffuse into water compared to shikonin.


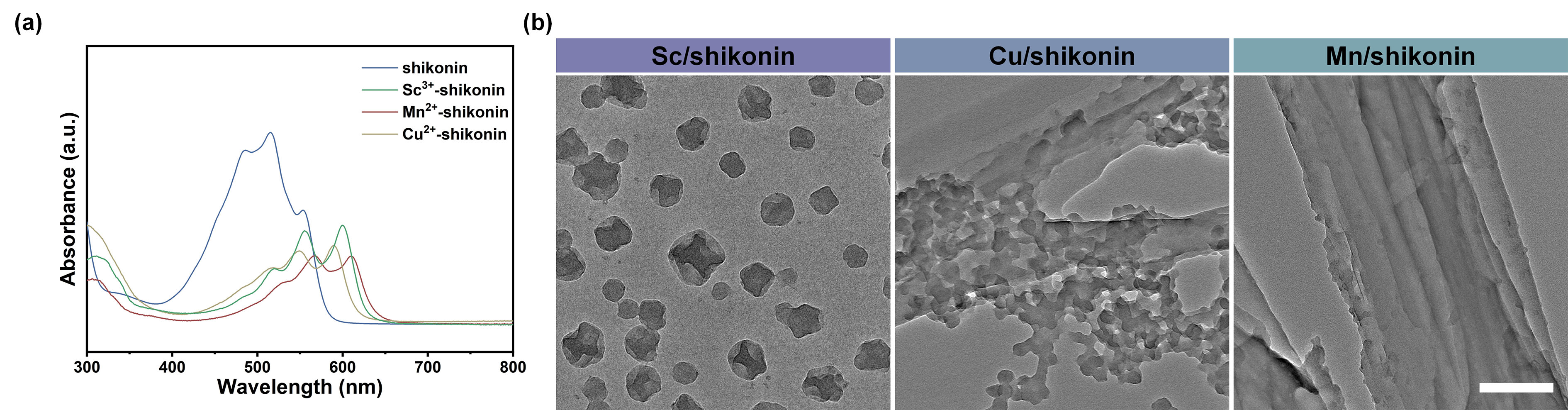


**Figure S9.** (a) UV-vis absorption spectra of Sc^3+^-shikonin, Cu^2+^-shikonin, and Mn^2+^-shikonin complexes in ethanol. (b) TEM images of Sc/shikonin, Cu/shikonin, and Mn/shikonin assemblies. The scale bar is 200 nm. The highest absorbance of Sc^3+^-shikonin complex indicates the strongest coordination interaction, which is helpful for forming compact interfacial networks and non-symmetric Sc/shikonin nanoarchitectonics. As for Cu^2+^ and Mn^2+^ with weaker coordination ability, the interfacial networks are not robust enough for diffusion inhibition, and both nanoarchitectonics and micro-ribbons are obtained. Note that the coordination interaction of shikonin with Sc^3+^, Cu^2+^ and Mn^2+^ is much weaker than that with Fe^3+^.^[6]^ So, the relatively strong coordination facilitates the formation of compact interfacial networks.


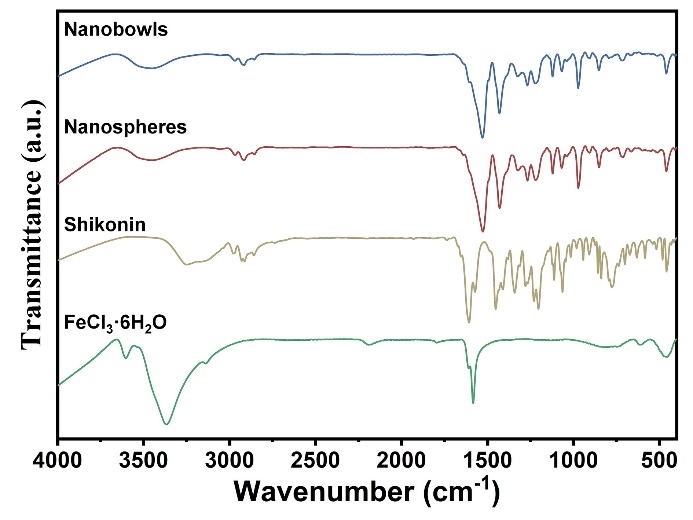


**Figure S10.** FTIR spectra of nanobowls, nanospheres, shikonin and FeCl_3_·6H_2_O.


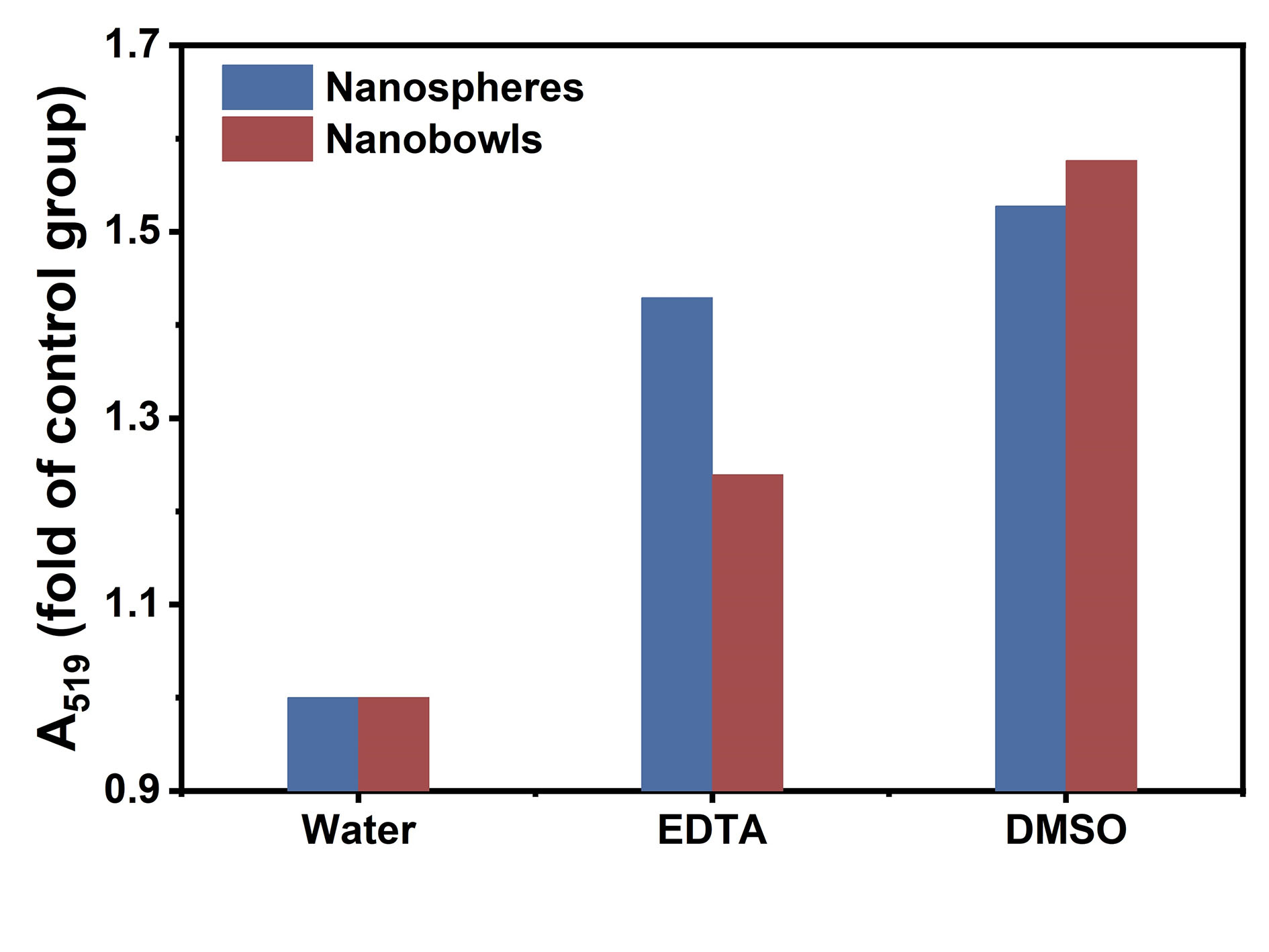


**Figure S11.** Variation of A_519_ of nanospheres and nanobowls after the treatment with water, EDTA, and DMSO for 6 h. According to the previous work,^[7]^ A_519_ represents released shikonin molecules. The water-treated group is set as control group.

**Table S2.** Composition analyses of Fe/shikonin nanoarchitectonics *via* ICP-AES.

| Nanoarchitectonics | Component | Molar content (%) |
| --- | --- | --- |
| Fe/shikonin nanospheres | Fe | 27.5 |
|  | Shikonin | 72.5 |
| Fe/shikonin nanobowls | Fe | 26.5 |
|  | Shikonin | 73.5 |


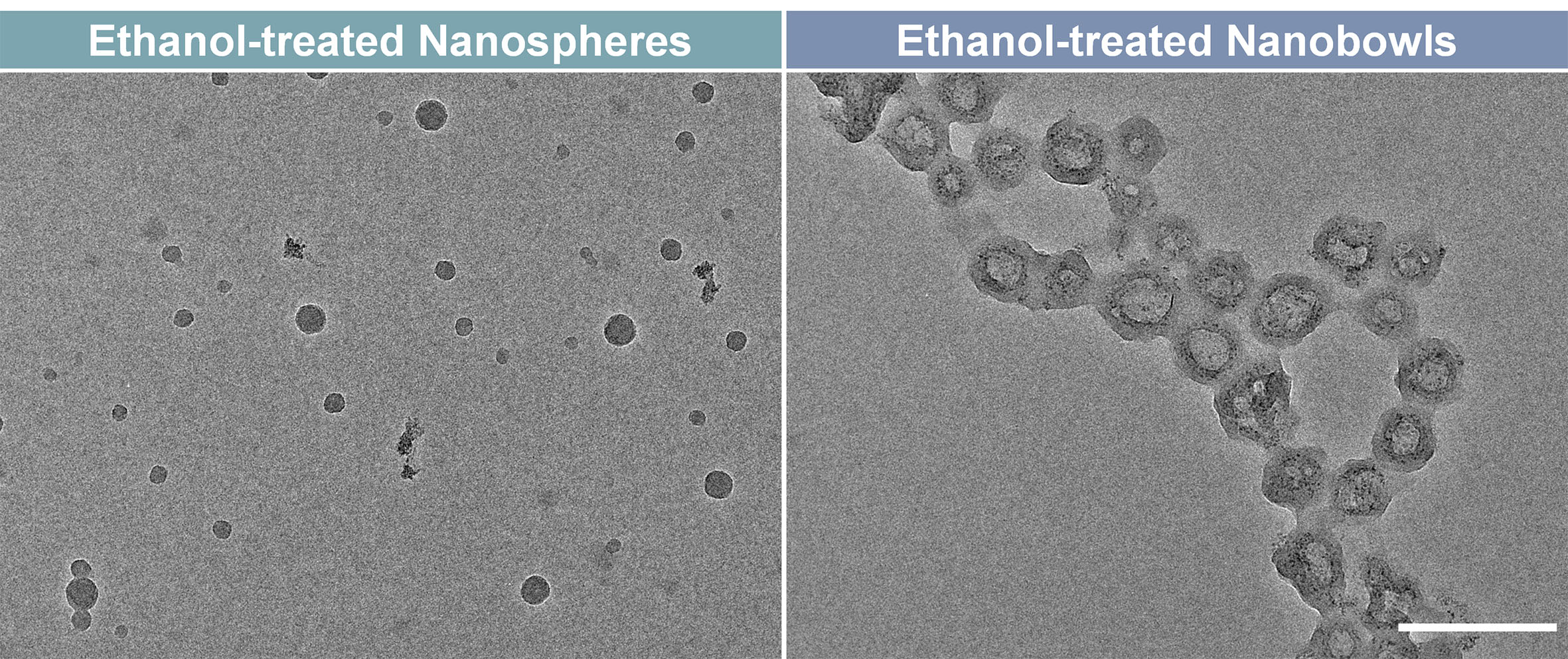


**Figure S12.** TEM images of nanospheres and nanobowls after the treatment with ethanol for 6 h. The scale bar is 200 nm.


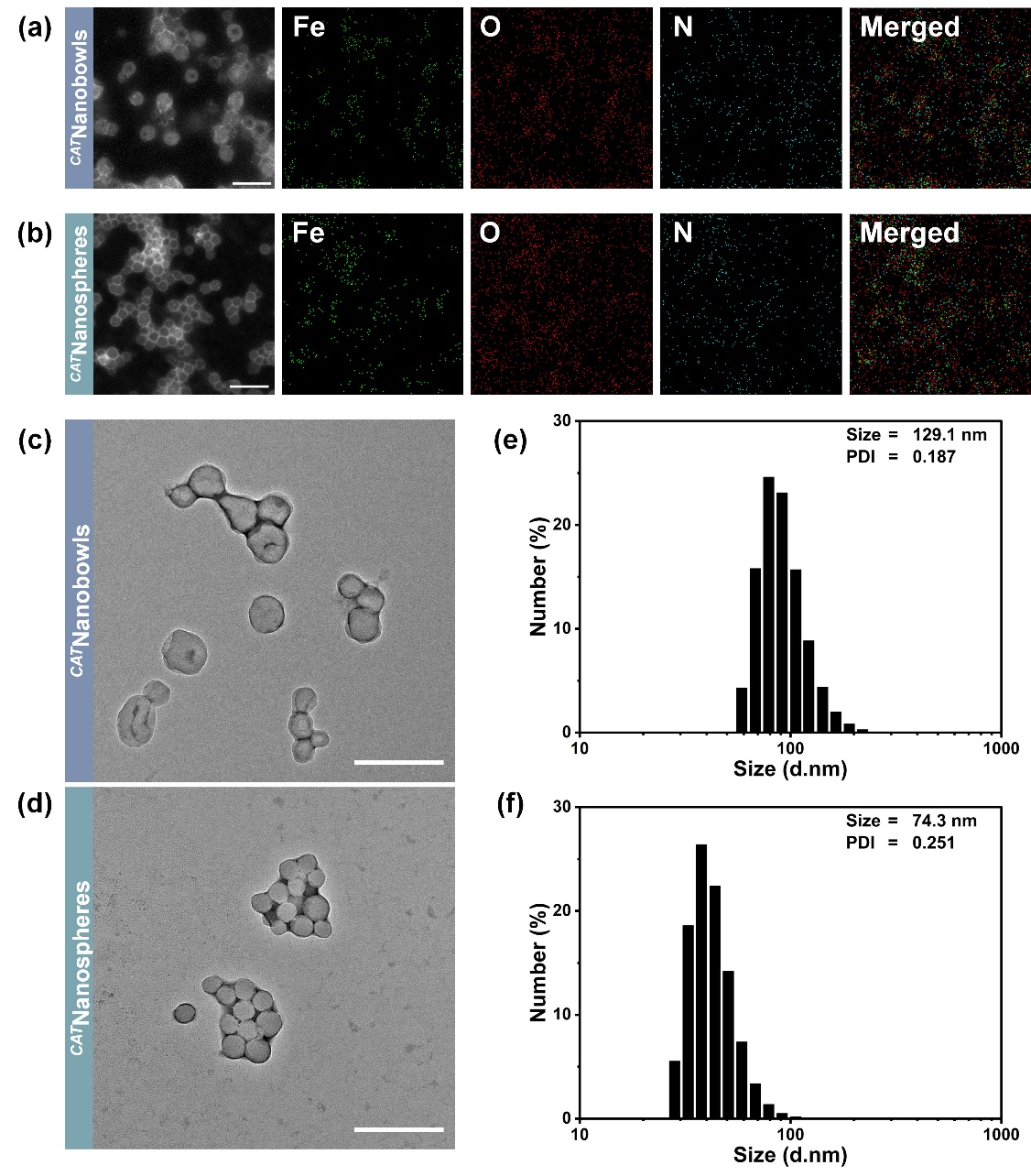


**Figure S13.** TEM-mapping images of *^CAT^*nanobowls (a) and *^CAT^*nanospheres (b). Negative-stained TEM images of *^CAT^*nanobowls (c) and *^CAT^*nanospheres (d). The scale bars are 200 nm. Hydrodynamic diameters of *^CAT^*nanobowls (e) and *^CAT^*nanospheres (f).


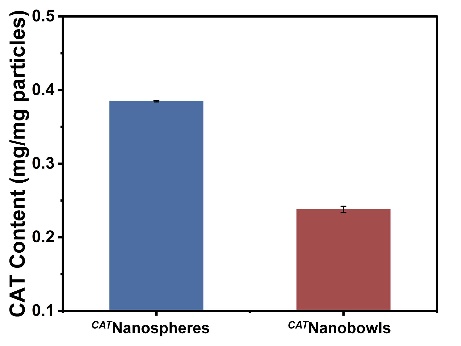


**Figure S14.** CAT content of *^CAT^*nanospheres and *^CAT^*nanobowls determined by BCA protein assay kit (n=3). Data are shown as mean ± SD and n represents the number of independent samples.


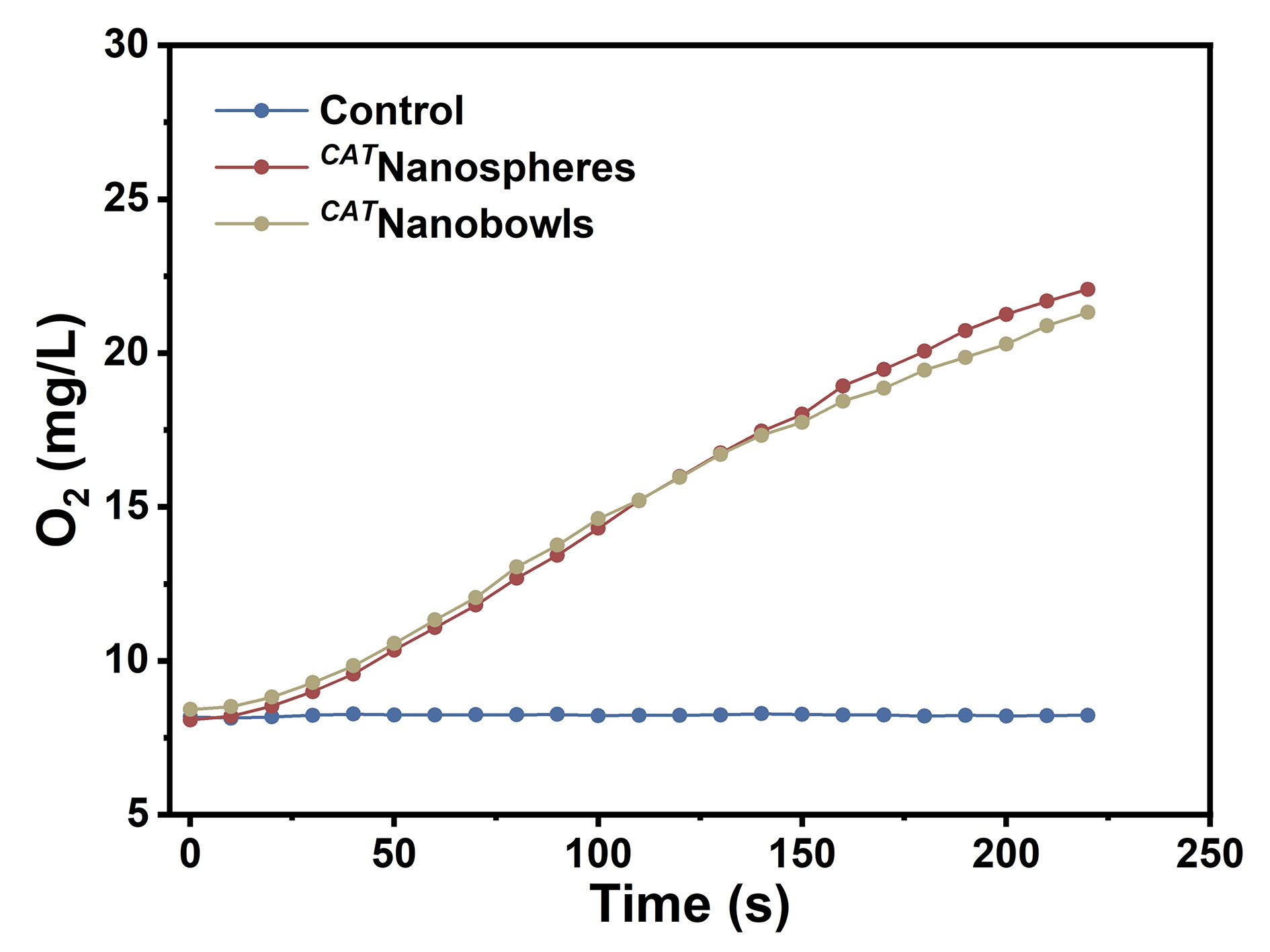


**Figure S15.** O_2_ generation in 10 mM H_2_O_2_ aqueous solution in the presence of *^CAT^*nanospheres and *^CAT^*nanobowls.


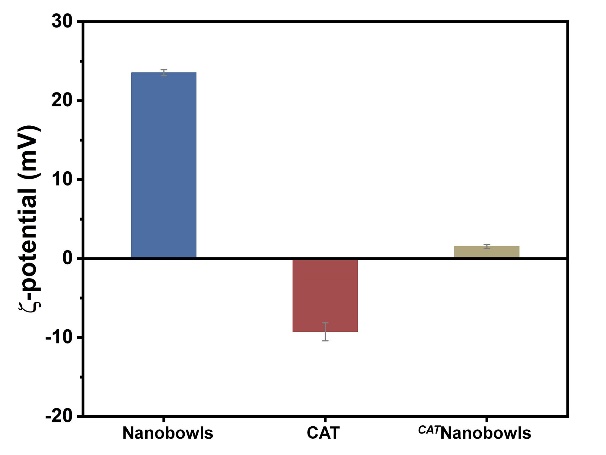


**Figure S16.** ζ-Potential of nanobowls, CAT and *^CAT^*nanobowls (n=3). Data are shown as mean ± SD and n represents the number of independent samples.


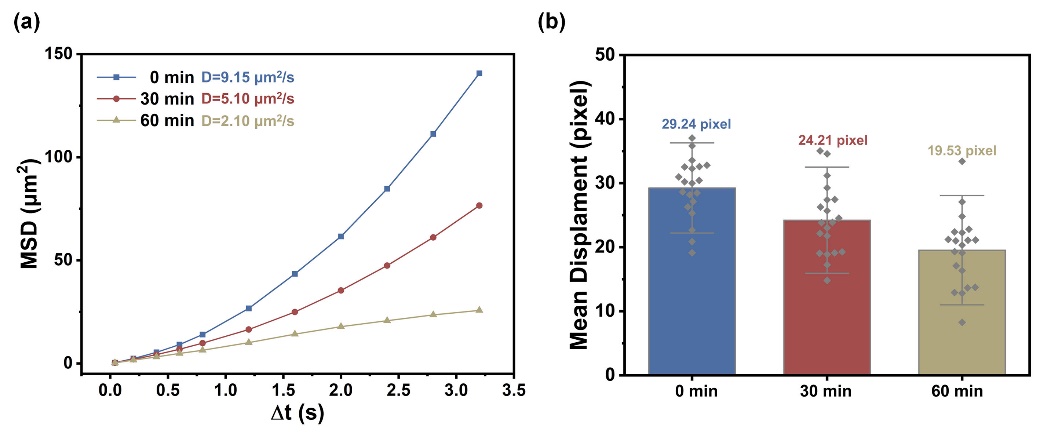


**Figure S17.** MSD curve (a) and mean displacement (b) of *^CAT^*nanobowls in 100 mM H_2_O_2_ aqueous solution at 0, 30 and 60 min (n=20). Data are shown as mean ± SD and n represents the number of independent samples.


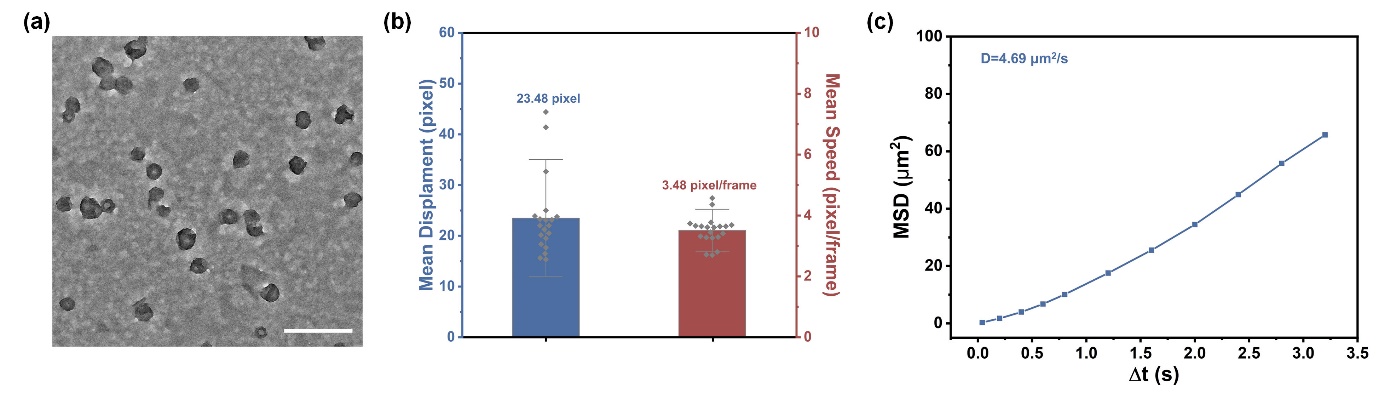


**Figure S18.** (a) TEM images of *^CAT^*nanobowls after immersing in cell medium for 24 h. Scale bar is 500 nm. (b) Mean displacement and mean speed of *^CAT^*nanobowls in cell medium (n=20). Data are shown as mean ± SD and n represents the number of independent samples. (c) MSD curve of *^CAT^*nanobowls in cell medium.


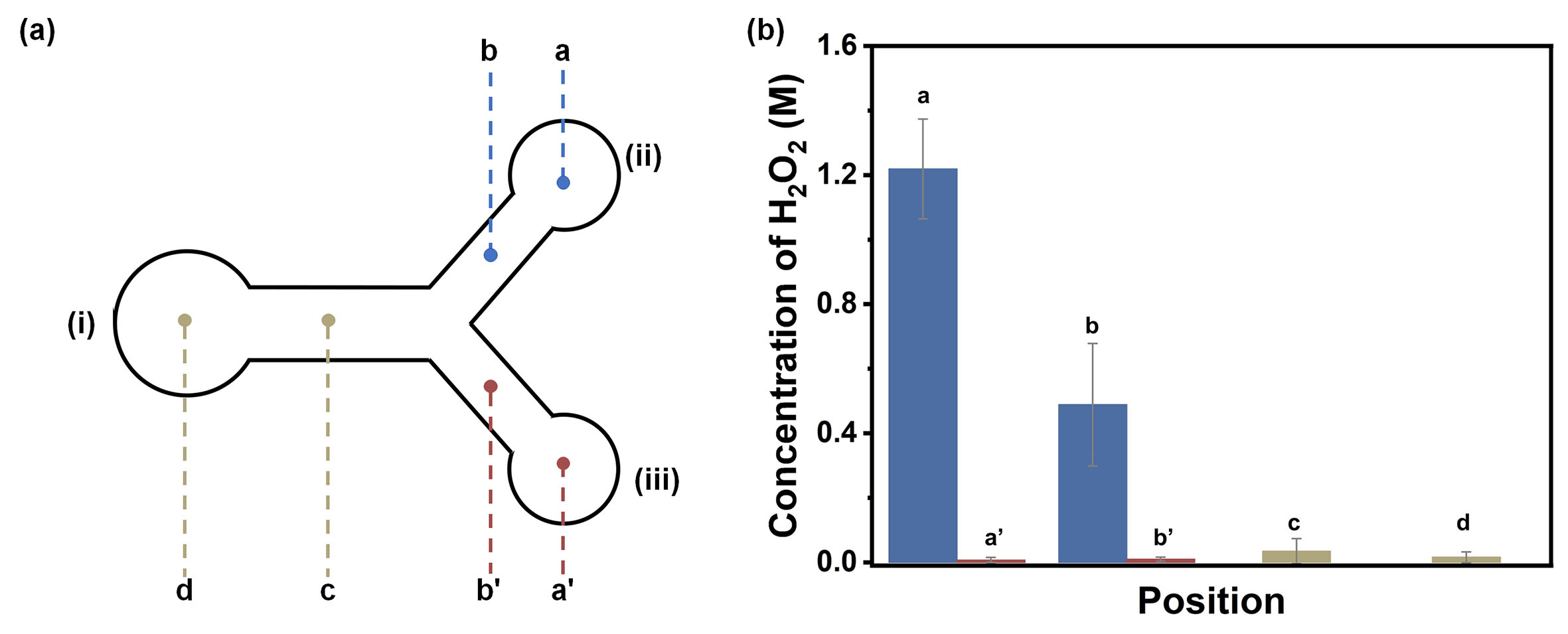


**Figure S19.** (a) Schematic illustration of Y-shaped device, where nanoarchitectonics, H_2_O_2_-contained agarose, and H_2_O-contained agarose are in reservoir (i), (ii), and (iii), respectively. (b) Concentration of H_2_O_2_ at specific point in Y-shaped device as indicated in (a) (n=3). Data are shown as mean ± SD and n represents the number of independent samples.


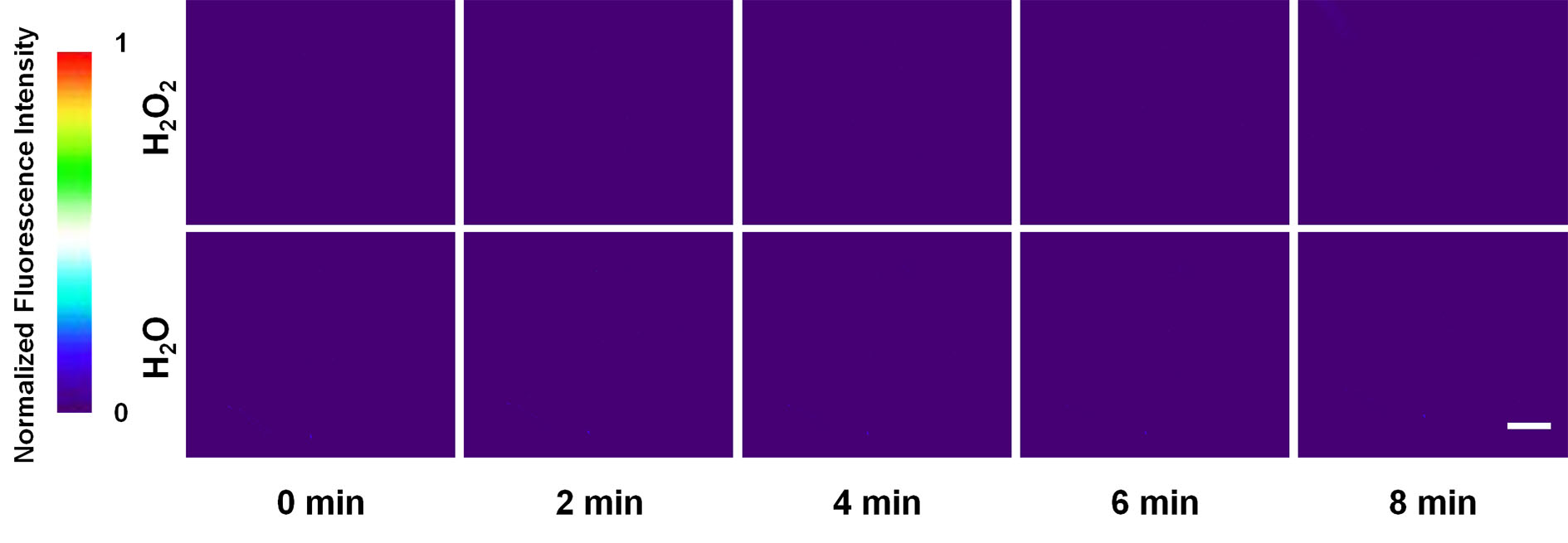


**Figure S20.** Normalized fluorescence quantitative images of *^CAT^*nanospheres in reservoir (ii) and (iii) of Y-shaped device. The scale bar is 1000 μm.


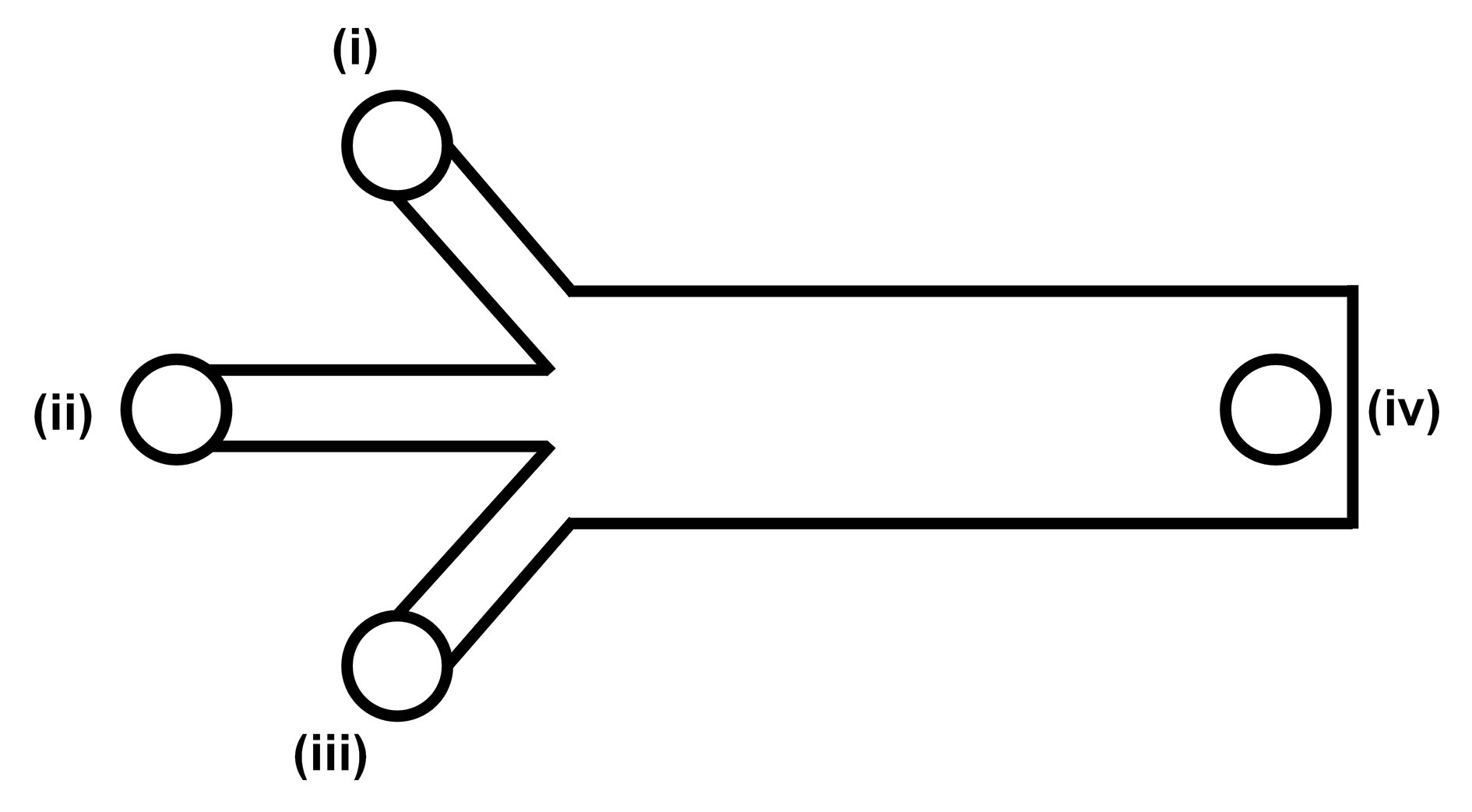


**Figure S21.** Schematic illustration of three-inlet one-outlet Ψ-shaped microfluidic device, where H_2_O_2_, nanoarchitectonics, and H_2_O flow into device from inlet (i), (ii), and (iii), respectively, then flow out from outlet (iv).


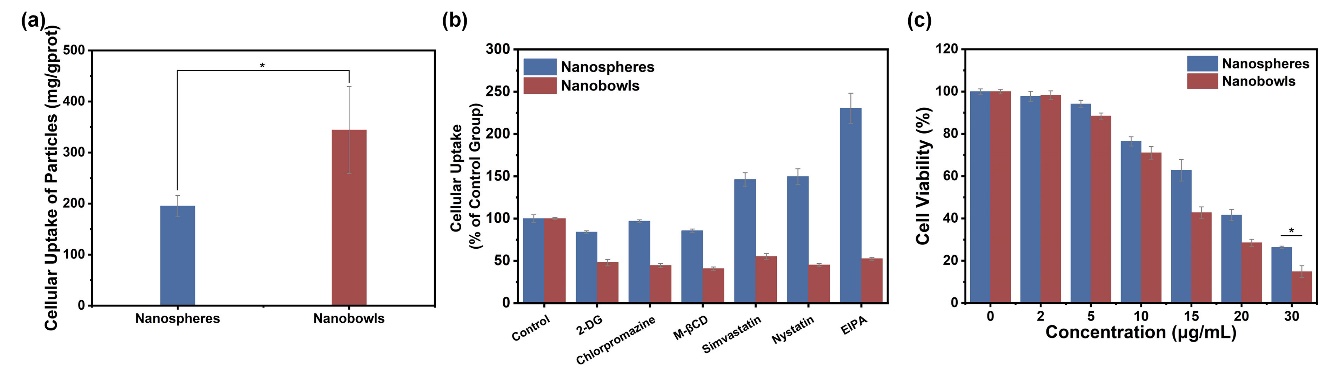


**Figure S22.** (a) Cellular uptake of nanospheres and nanobowls in 4T1 cells after incubation for 6 h (n=3). (b) Cellular uptake of nanospheres and nanobowls in 4T1 cells analyzed by flow cytometry using different endocytosis inhibitors, including energy-dependent endocytosis inhibitor 2-DG, clathrin-dependent endocytosis inhibitors chlorpromazine and M-βCD, caveolin-dependent simvastatin and nystatin, and micropinocytosis inhibitor EIPA (n=3). (c) Cell viability of 4T1 cells after incubation with specific concentration of nanospheres and nanobowls for 24 h (n=5). Data are shown as mean ± SD and n represents the number of biologically independent samples. * P<0.05.


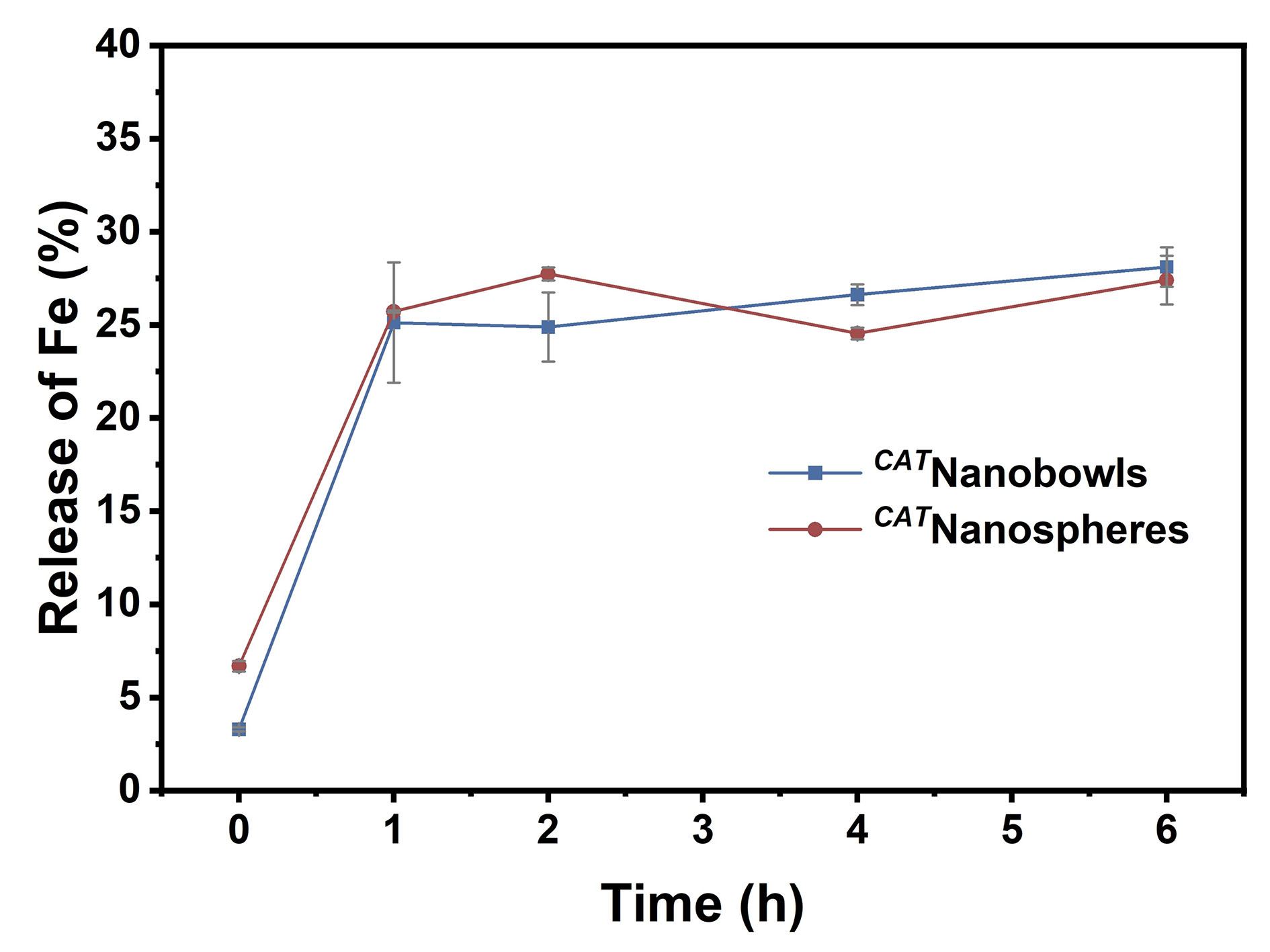


**Figure S23.** Accumulative Fe^2+^ release of *^CAT^*nanospheres and *^CAT^*nanobowls in the presence of 10 mM GSH (n=3). Data are shown as mean ± SD and n represents the number of independent samples. In the simulated condition, GSH reduces Fe^3+^ to Fe^2+^, leading to the breakdown of the Fe/shikonin coordination network and the concomitant release of Fe^2+^ and free shikonin. Due to its limited solubility, the liberated shikonin tends to precipitate from solution. As both residual Fe^3+^ and the newly formed Fe^2+^ can still coordinate with shikonin, this precipitation results in the co-removal of Fe species from the supernatant. Consequently, the detectable Fe^2+^ concentration in solution reaches equilibrium within 1 hour.

**
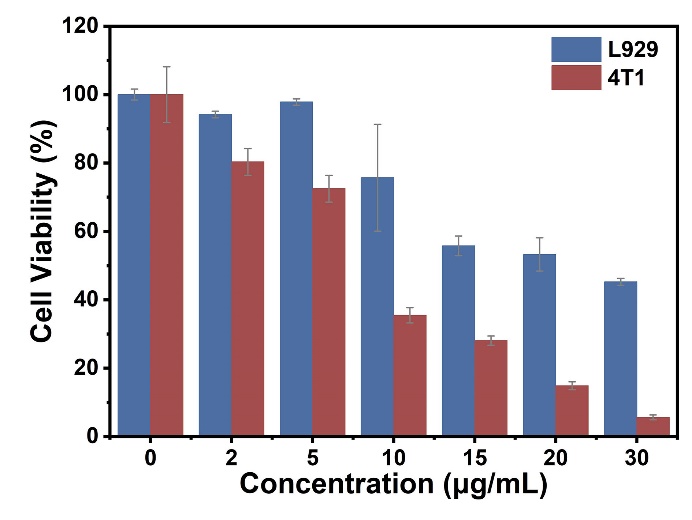
**

**Figure S24.** Cell viability of 4T1 and L929 cells after incubation with specific concentration of *^CAT^*nanobowls for 24 h (n=5). Data are shown as mean ± SD and n represents the number of biologically independent samples.


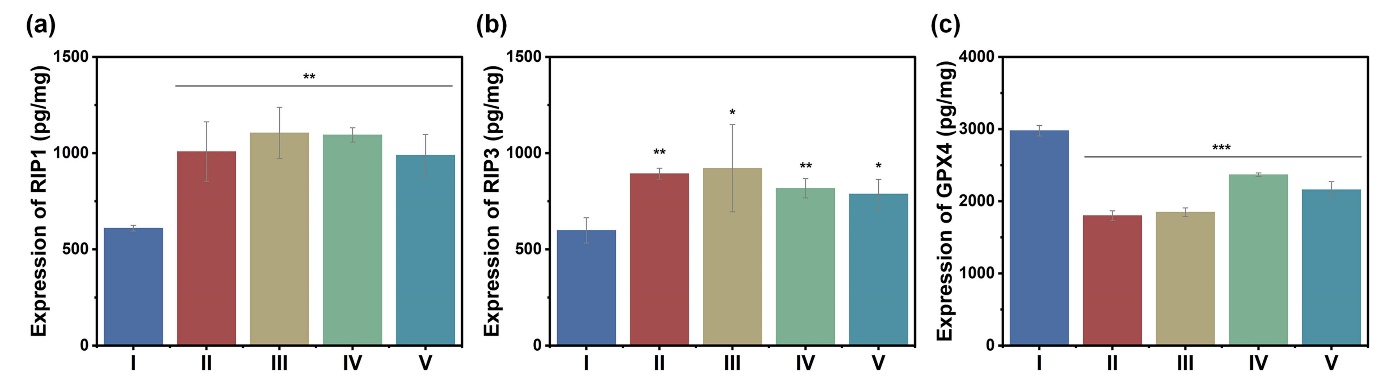


**Figure S25.** Expression of RIP1 (a), RIP3 (b), and GPX4 (c) of 4T1 cells analyzed by ELISA assay kit after treatment with medium (I), 30 μg/mL nanospheres (II), nanobowls (III), *^CAT^*nanospheres (IV), and *^CAT^*nanobowls (V) for 24 h (n=3). Data are shown as mean ± SD and n represents the number of biologically independent samples. * p < 0.05, ** p < 0.01, *** p < 0.001.


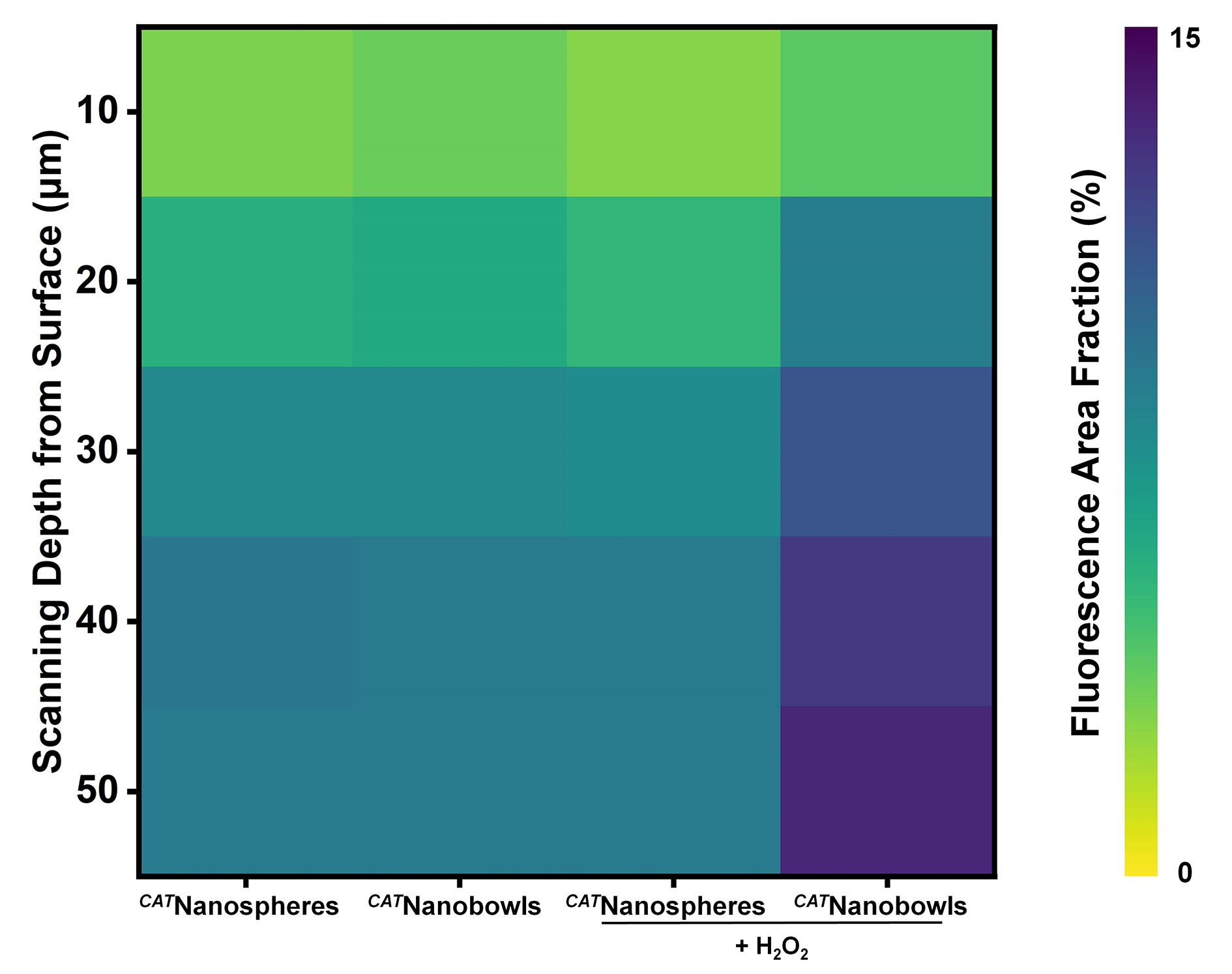


**Figure S26.** Heatmap of fluorescence area fraction of z-stacking CLSM images of 3D MTSs with different treatments.


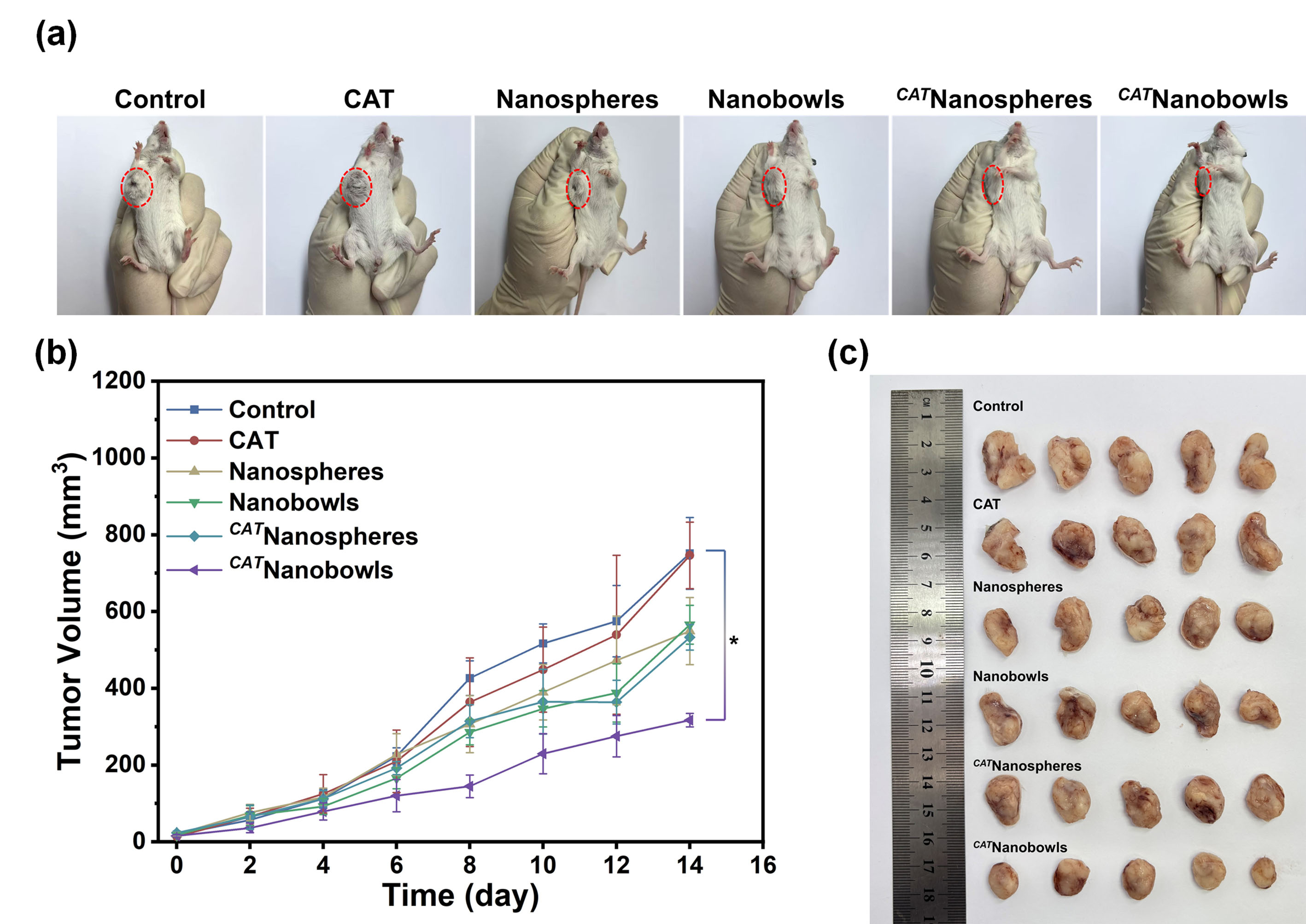


**Figure S27.** (a) Representative images of 4T1 tumor-bearing mice after different treatments for 14 days. (b) Tumor volume of mice in each group throughout the treatment period (n=5). (c) Photograph of tumors in each group. Data are shown as mean ± SD and n represents the number of biologically independent samples. * P<0.05.

**
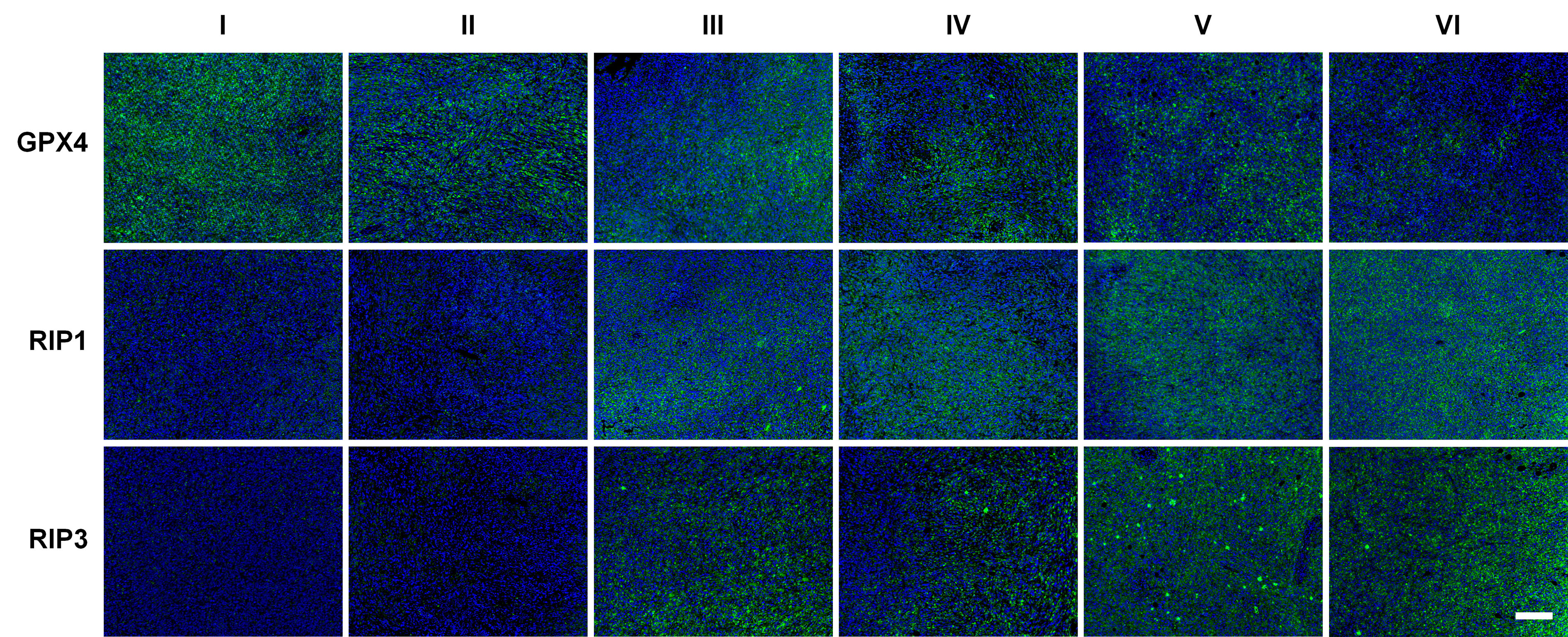
**

**Figure S28.** Representative GPX4, RIP1 and RIP3 immunofluorescence staining images of tumors collected from mice in control (I), CAT (II), nanosphere (III), nanobowl (IV), *^CAT^*nanosphere (V), and *^CAT^*nanobowl (VI) groups after different treatment for 14 days. The scale bar is 200 μm.


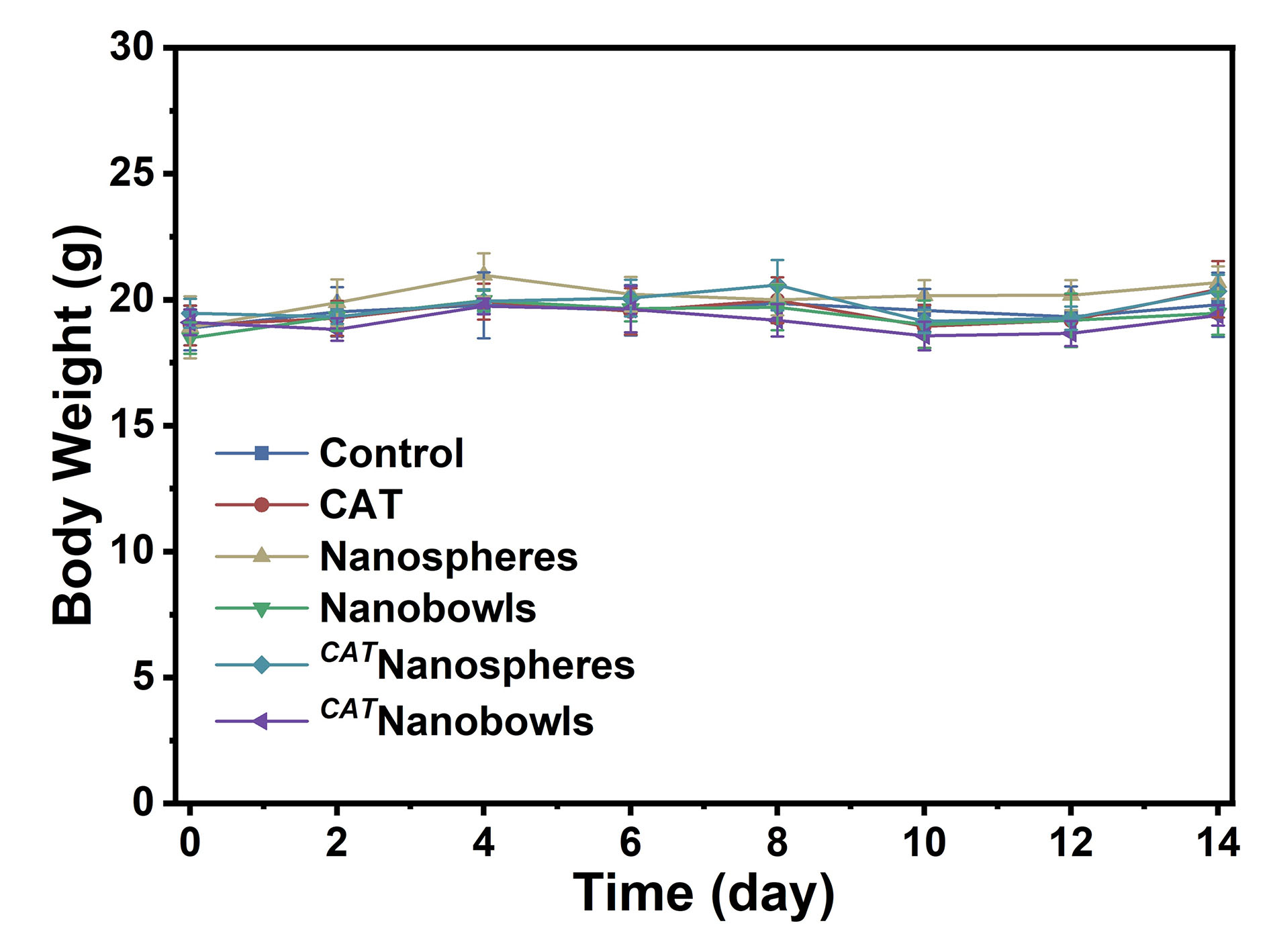


**Figure S29.** The body weight of mice in each group throughout the treatment period (n=5). Data are shown as mean ± SD and n represents the number of biologically independent samples.


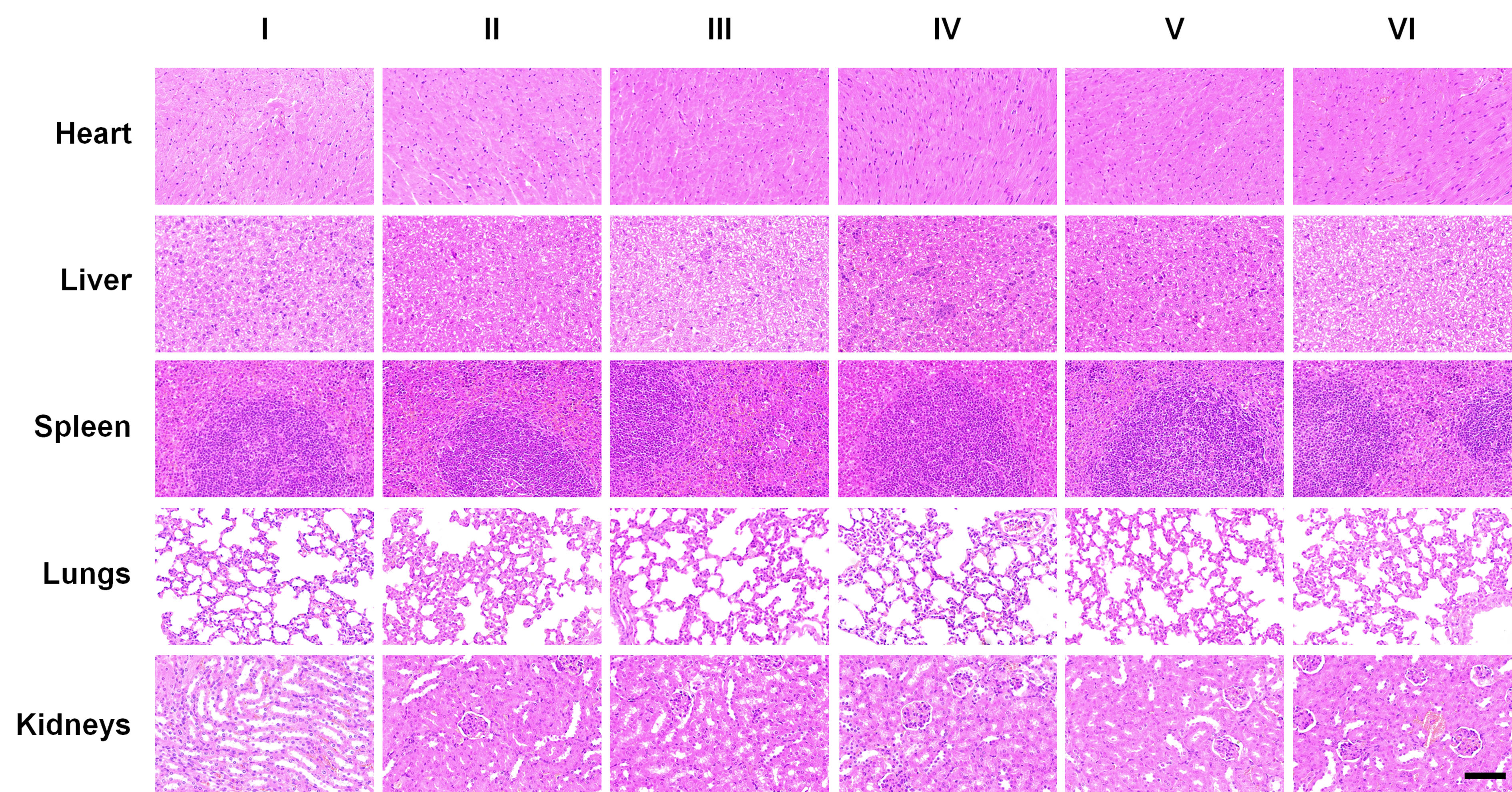


**Figure S30.** Representative H&E staining images of heart, liver, spleen, lungs, and kidneys of mice in control (I), CAT (II), nanosphere (III), nanobowl (IV), *^CAT^*nanosphere (V), and *^CAT^*nanobowl (VI) groups after different treatment for 14 days. The scale bar is 100 μm.


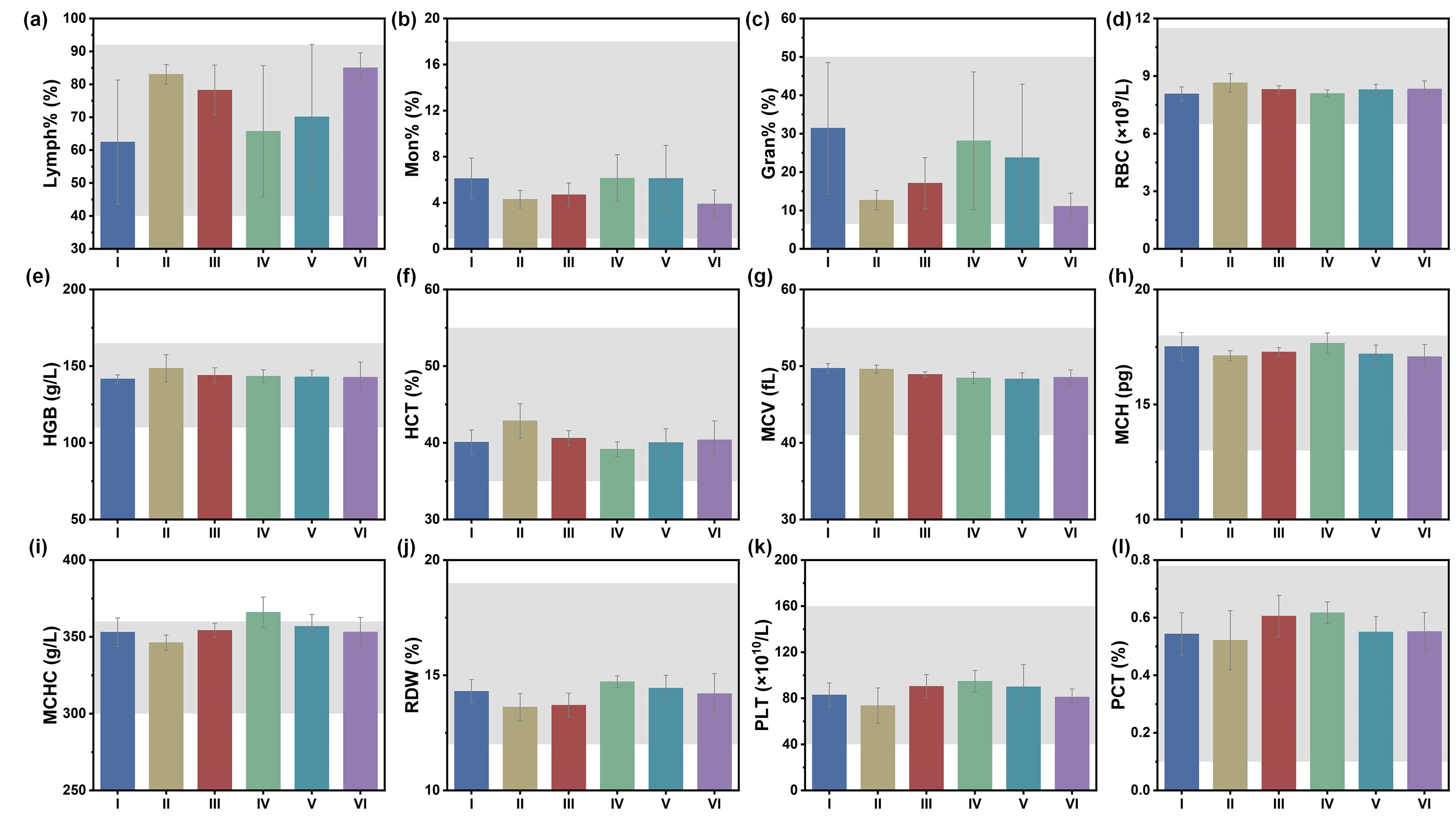


**Figure S31.** Blood routine examinations of the mice in control (I), CAT (II), nanosphere (III), nanobowl (IV), *^CAT^*nanosphere (V), and *^CAT^*nanobowl groups. (a) lymphocyte ratio (Lymph%), (b) monocyte ratio (Mon%), (c) gran ratio (gran%), (d) red blood cell count (RBC), (e) hemoglobin (HGB), (f) hematocrit (HCT), (g) mean corpuscular volume (MCV), (h) mean corpuscular hemoglobin (MCH), (i) mean corpuscular hemoglobin concentration (MCHC), (j) red cell distribution width (RDW), (k) platelet (PLT), and (l) plateletcrit (PCT). Data are shown as mean ± SD. n = 5, n represents the number of biologically independent samples.

**Supplementary Movie List**

**Movie S1.** Nanospheres moving in 100 mM H_2_O_2_ aqueous solution.

**Movie S2.** Nanobowls moving in 100 mM H_2_O_2_ aqueous solution.

**Movie S3.** *^CAT^*Nanospheres moving in 100 mM H_2_O_2_ aqueous solution.

**Movie S4.** *^CAT^*Nanobowls moving in 100 mM H_2_O_2_ aqueous solution.

**Movie S5.** *^CAT^*Nanobowls moving in 0 mM H_2_O_2_ aqueous solution.

**Movie S6.** *^CAT^*Nanobowls moving in 10 mM H_2_O_2_ aqueous solution.

**Movie S7.** *^CAT^*Nanobowls moving in 100 mM H_2_O_2_ aqueous solution.

**Movie S8.** *^CAT^*Nanobowls moving in 1000 mM H_2_O_2_ aqueous solution.

References

1. (a) Pijpers, I. A. B., Cao, S., Llopis-Lorente, A., Zhu, J., Song, S., Joosten, R. R. M., Meng, F., Friedrich, H, Williams, D. S., Sánchez, S., van Hest, J. C. M., Abdelmohsen, L. K. E. A. Hybrid biodegradable nanomotors through compartmentalized synthesis. *Nano Lett.* **2020**, *20*, 4472-4480; (b) Serra-Casablancas, M., Di Carlo, V., Esporrín-Ubieto, D., Prado-Morales, C., Bakenecker, A. C., Sánchez, S. Catalase-powered nanobots for overcoming the mucus barrier. *ACS Nano* **2024**, *18*, 16701-16714; (c) Liu, M., Chen, L., Zhao, Z., Liu, M., Zhao, T., Ma, Y., Zhou, Q., Ibrahim, Y. S., Elzatahry, A. A., Li, X., Zhao, D. Enzyme-based mesoporous nanomotors with near-infrared optical brakes. *J. Am. Chem. Soc.* **2022**, *144*, 3892-3901.
2. Li, T., Liu, Z., Hu, J., Chen, L., Chen, T., Tang, Q., Yu, B., Zhao, B., Mao, C., Wan, M. A universal chemotactic targeted delivery strategy for inflammatory diseases. *Adv. Mater.* **2022**, *34*, 2206654.
3. Pyeon, J., Song, K. M., Jung, Y. S., Kim, H. Self-induced solutal marangoni flows realize coffee-ring-less quantum dot microarrays with extensive geometric tunability and scalability. *Adv. Sci.* **2022**, *9*, 2104519.
4. Haynes, W. M. *CRC Handbook of Chemistry and Physics (97th ed.)* *Section 6*, Boca Raton, 2016, pp. 1-262.
5. Liu, S., Zhang, M., Jin, H., Wang, Z., Liu, Y., Zhang, S., Zhang, H. Iron-containing protein-mimic supramolecular iron delivery systems for ferroptosis tumor therapy. *J. Am. Chem. Soc.* **2023**, *145*, 160-170.
6. Dean, J. A. *Lange’s Handbook of Chemistry (15th ed) Section 8,* McGraw-Hill, New York, 1999, pp. 82-88.
7. Xu, W., Chen, Y., Yang, R., Fu, Y., Zhuang, W., Wang, Y., Liu, Y., Zhang, H. “Reaction”-like shaping of self-delivery supramolecular nanodrugs in the nanoprecipitation process. *ACS Nano* **2023**, *17*, 18227-18239.
